# LANA oligomeric architecture is essential for KSHV nuclear body formation and viral genome maintenance during latency

**DOI:** 10.1371/journal.ppat.1007489

**Published:** 2019-01-25

**Authors:** Alessandra De Leo, Zhong Deng, Olga Vladimirova, Horng-Shen Chen, Jayaraju Dheekollu, Abram Calderon, Kenneth A. Myers, James Hayden, Frederick Keeney, Benedikt B. Kaufer, Yan Yuan, Erle Robertson, Paul M. Lieberman

**Affiliations:** 1 Program in Gene Expression and Regulation, The Wistar Institute, Philadelphia, Pennsylvania, United States of America; 2 Department of Biological Sciences, University of the Sciences, Philadelphia, Pennsylvania, United States of America; 3 Department of Virology, Institute Virology, Freie Universitat Berlin, Berlin, Germany; 4 Department of Biochemistry, School of Dentistry, University of Pennsylvania, Philadelphia, Pennsylvania, United States of America; 5 Department of Microbiology, Perelman School of Medicine at the University of Pennsylvania, Philadelphia, Pennsylvania, United States of America; Harvard University, UNITED STATES

## Abstract

The molecular basis for the formation of functional, higher-ordered macro-molecular domains is not completely known. The Kaposi’s Sarcoma-Associated Herpesvirus (KSHV) genome forms a super-molecular domain structure during latent infection that is strictly dependent on the DNA binding of the viral nuclear antigen LANA to the viral terminal repeats (TR). LANA is known to form oligomeric structures that have been implicated in viral episome maintenance. In this study, we show that the LANA oligomerization interface is required for the formation of higher-order nuclear bodies that partially colocalize with DAXX, EZH2, H3K27me3, and ORC2 but not with PML. These nuclear bodies assemble at the periphery of condensed cellular chromosomes during mitotic cell division. We demonstrate that the LANA oligomerization interface contributes to the cooperative DNA binding at the viral TR and the recruitment of ORC to the viral episome. Oligomerization mutants failed to auto-regulate LANA/ORF73 transcription, and this correlated with the loss of a chromosome conformational DNA-loop between the TR and LANA promoter. Viral genomes with LANA oligomerization mutants were subject to genome rearrangements including the loss of subgenomic DNA. Our data suggests that LANA oligomerization drives stable binding to the TR and formation of an epigenetically stable chromatin architecture resulting in higher-order LANA nuclear bodies important for viral genome integrity and long-term episome persistence.

## Author summary

KSHV genomes persist in large nuclear bodies in latently infected cells. The KSHV encoded nuclear antigen LANA is required for the efficient replication and stable maintenance of viral genomes during latent infection. LANA is also known to form oligomeric structures, but it is not known how these structures contribute to LANA function in living cells. Here, we show that LANA oligomerization is required for cooperative binding to the KSHV terminal repeat (TR), and the recruitment of the Origin Recognition Complex (ORC) to viral TR. LANA oligomerization is required for a chromosome conformation DNA loop between TR and the LANA promoter implicated in LANA transcription autoregulation. LANA oligomerization is also required for formation of large nuclear bodies that colocalize with DAXX, EZH2, ORC2, but not PML. LANA nuclear bodies distribute along the nuclear periphery, and their arrangement is transmitted faithfully to daughter cells during mitotic cell division. Finally, we show that KSHV genomes containing mutations in the LANA oligomerization interface fail to maintain the complete viral genome, suggesting they are defective in DNA replication or repair. These findings reveal new mechanisms of LANA episome maintenance through formation of higher-order chromosome-conformations.

## Introduction

Kaposi’s Sarcoma Associated Herpesvirus (KSHV) is a human gammaherpesvirus responsible for Kaposi’s Sarcoma (KS), Pleural Effusion Lymphoma (PEL), and multicentric Castleman’s Disease (mCD) (reviewed in [1, 2]). KSHV is a large double-stranded DNA virus that establishes life-long latent infection in B-lymphocytes and potentially other cell types, such as mesenchymal stem cells and endothelial cells. Most KSHV-associated cancers harbor latent viral genomes in the nucleus of tumor cells. The latent genomes form covalently closed circular genomes, termed episomes, that establish a local chromatin structure similar to that of cellular genomic DNA (reviewed in [3–5]). During latency, viral gene expression is restricted to a small set of viral genes. Viral DNA replication occurs coordinately with host cell division cycle utilizing host cellular DNA replication machinery.

Stable maintenance of the latent episome requires the regulated assembly of chromatin factors on the viral genome to enable selective expression of latent, but not lytic viral genes. Furthermore, the viral episome must replicate on average once per cell cycle and be transmitted faithfully to each daughter cell to maintain stable copy number in each daughter cell. The KSHV encoded Latency Associated Nuclear Antigen (LANA) is a multifunctional DNA binding protein that is essential for stable maintenance of KSHV episomes during latent infection [6–8]. The LANA DNA binding domain shares structural homology to the DNA binding domains of EBV EBNA1 and HPV E2 proteins ([9–12] reviewed in [13]). LANA binds directly to three recognition sites in the terminal repeats (TR) of the KSHV genome [9]. These sites represent a minimal origin of plasmid DNA replication [14], and at least two or more TRs are required for episome maintenance [15]. LANA is required for the efficient recruitment of cellular replication initiation factors, such as the Origin Recognition Complex (ORC) and the Mini Chromosome Maintenance (MCM) complex to the viral TR region [16, 17]. LANA binding to the TR is thought to influence the chromatin organization of the viral episome through recruitment of ORC and other chromatin-regulatory proteins [16, 18, 19]. LANA can also interact with core histones H2A/H2B through its amino terminal domain that facilitates attachment of the virus genome to a metaphase chromosome and is essential for viral genome transmission during cell division [20–23].

LANA is also thought to influence the higher-order chromatin and chromosomal structure of the KSHV episome during latency. Fluorescent light microscopy reveals that LANA forms large aggregate structures, often termed LANA speckles, that colocalize with KSHV episomes in the nucleoplasm during interphase and with telomeric and centromeric regions of the metaphase chromosome in mitosis [11, 15, 24, 25]. Some of these LANA structures were found to colocalize with heterochromatin-associated proteins, such as DAXX [26, 27] and the Polycomb-associated histone H3K27 methyltransferase EZH2 [28]. Super resolution imaging studies indicate that LANA coordinates a higher-ordered architecture at the TR of stable episomes [29]. Live cell imaging studies indicate that KSHV episomes can form large, multigenome clusters connected through LANA bivalent C-terminal DNA binding and N-terminal histone tethering activities [30, 31]. The LANA DNA binding domain (DBD) by itself can form a variety of higher-order oligomeric structures, as determined by X-ray crystallography and biochemical methods [10, 11]. Mutations in LANA that disrupt these higher order oligomers in vitro inhibited cooperative DNA binding and plasmid maintenance, but had no observable effect on DNA binding to single LANA binding sites [10]. It remains unclear whether molecular oligomerization of LANA DBD is directly associated with the higher order nuclear structures observed by microscopy methods [15, 24, 26, 27, 29, 30].

Here, we examine the role of the LANA DBD oligomerization interface in the assembly of these higher-order structures observed by fluorescence light-microscopy. In addition, we assessed if this process influences viral gene expression, chromosome conformation, and viral genome stability. Our data suggest that LANA DBD oligomerization provides the molecular basis for KSHV chromosome conformation and super-molecular nuclear bodies required for stable episome maintenance during latency.

## Results

### LANA forms nuclear bodies that colocalize with DAXX and EZH2, but not PML

**Fig 1:**
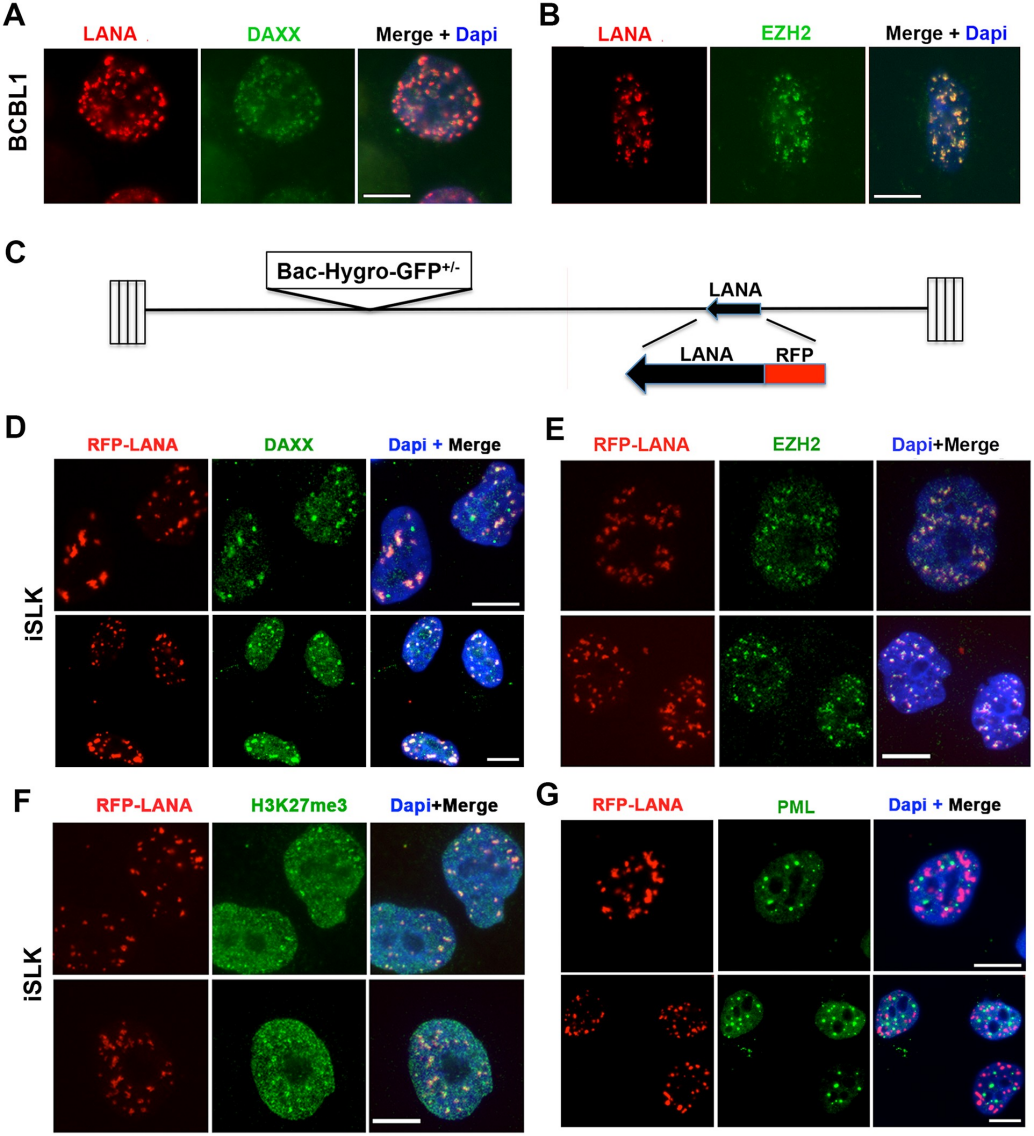
LANA forms nuclear bodies that colocalize with DAXX and EZH2, but not PML. **(A-B)** BCBL1 cells were imaged by indirect immunofluorescence with antibodies to LANA (red) and **(A)** DAXX (green), or **(B)** EZH2 (green) or merge with Dapi (blue). Scale bar = 10 μm. **(C)** Schematic of the RFP-LANA fusion integrated into the endogenous LANA of the KSHV BAC16 genome. **(D-G)** Stable iSLK cells with BAC16 expressing RFP-LANA (red) were imaged by indirect immunofluorescence with antibodies to **(D)** DAXX (green), **(E)** EZH2 (green), **(F)** H3K27me3 (green), or **(G)** PML (green). Cells were counter-stained with Dapi (blue) as shown in merge. Scale bar = 10 μm.

LANA has been shown to form multiple aggregate structures of various sizes in the nucleus of latently infected cells [11, 15, 24, 25]. These structures have also been shown to colocalize with several other nuclear factors, including DAXX [26] and EZH2 [28]. We confirmed these findings in BCBL1 pleural effusion lymphoma cell line (Fig 1A and 1B). To further investigate the formation and transmission of these LANA structures in living cells, we fused an amino-terminal RFP to the N-terminus of LANA in the infectious KSHV bacterial artificial chromosome (BAC) clone BAC16 (Fig 1C). We generated stable iSLK cell lines carrying latent KSHV BAC16 genomes that constitutively express RFP-LANA. RFP-LANA in iSLK cells formed similar nuclear body patterns as was observed for endogenous LANA in BCBL1 cells, including the colocalization with DAXX (Fig 1D) and EZH2 (Fig 1E). We also found that RFP-LANA partially colocalizes with H3K27me3 (Fig 1F), consistent with the role of EZH2 in histone methylation. While DAXX commonly colocalizes with the anti-viral protein PML and PML-nuclear bodies (PML-NBs), we found that RFP-LANA did not colocalize with PML (Fig 1G). These findings indicate that RFP-LANA expressed from KSHV BAC16 in iSLK cells forms similar nuclear bodies to that observed in naturally occurring latently infected PEL cells, and that these LANA bodies are distinct from PML-NBs.

### LANA nuclear bodies remain stable and organized through cell division

**Fig 2:**
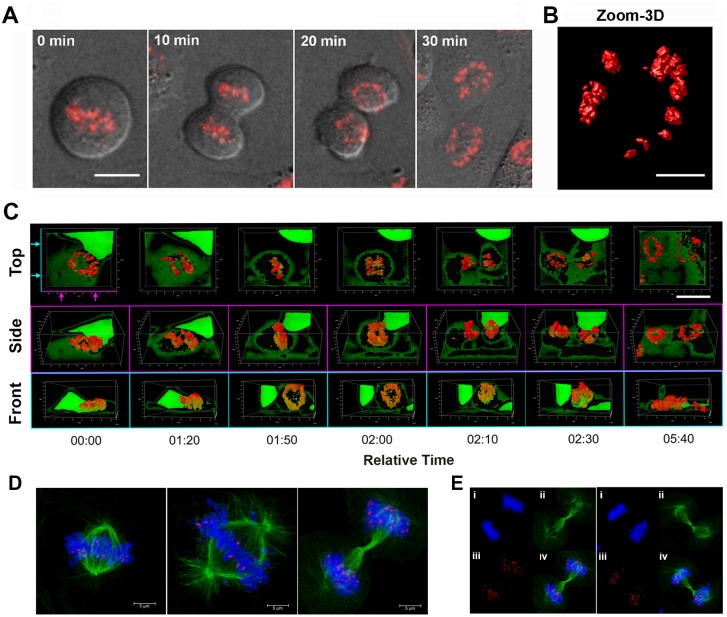
Dynamic organization of LANA nuclear bodies through mitotic cell division. **(A)** Live cell imaging of RFP-LANA in iSLK cell line combined with Phase contrast during a 30 min interval through mitotic cell division. Scale bar = 10 μm. **(B)** Zoom and 3D reconstruction of confocal images of LANA bodies formed during live cell imaging in iSLK cells. Scale bar = 5 μm. **(C)** Confocal images of RFP-LANA from 3 orthogonal viewpoints at intervals across cell division. Relative time (min:sec) is indicated below each frame. Scale bar = 20 μm. **(D)** Confocal fixed images of mitotic RFP-LANA (red), α-tubulin (green) and Dapi (blue) in iSLK cells. Scale bar = 5 μm. **(E)** Deconvolved confocal images as shown in panel D with Dapi (i), α-tubulin (ii), RFP-LANA (iii), and merge (iv).

To investigate the stability and transmission of LANA nuclear bodies through the cell cycle, we used live cell confocal microscopy of RFP-LANA in stable iSLK cells carrying KSHV BAC16 expressing RFP-LANA (Fig 2). During cell division, RFP-LANA bodies assemble on the metaphase plate and segregate with equal partitioning to daughter cells (Fig 2A). Within 20 min after metaphase LANA clusters reorganized in a distribution pattern strikingly similar to the parent cell, typically forming a ring of partially connected bodies along the nuclear periphery (Fig 2A). High resolution 3D reconstruction of the Z-stacked confocal images suggest that these nuclear bodies are heterogeneous in size (Fig 2B). Visualization of the division cycle from three different viewpoints revealed that the ring of LANA nuclear bodies undergoes an orthogonal re-orientation during telophase and the formation of two new rings in similar distribution and planar orientation as the parent cell (Fig 2C, supplemental movies **M1-2**). Costaining of mitotic figures with α-tubulin and Dapi further demonstrated that LANA bodies partition coordinately with host chromosomes with typical colocalization at the periphery of condensed metaphase chromosomes (Fig 2D and 2E). These findings indicate that LANA forms highly organized nuclear structures that undergo a coordinated transmission process and stable morphology in recipient daughter cells.

### LANA oligomerization interface is important for cooperative DNA binding to TR

**Fig 3:**
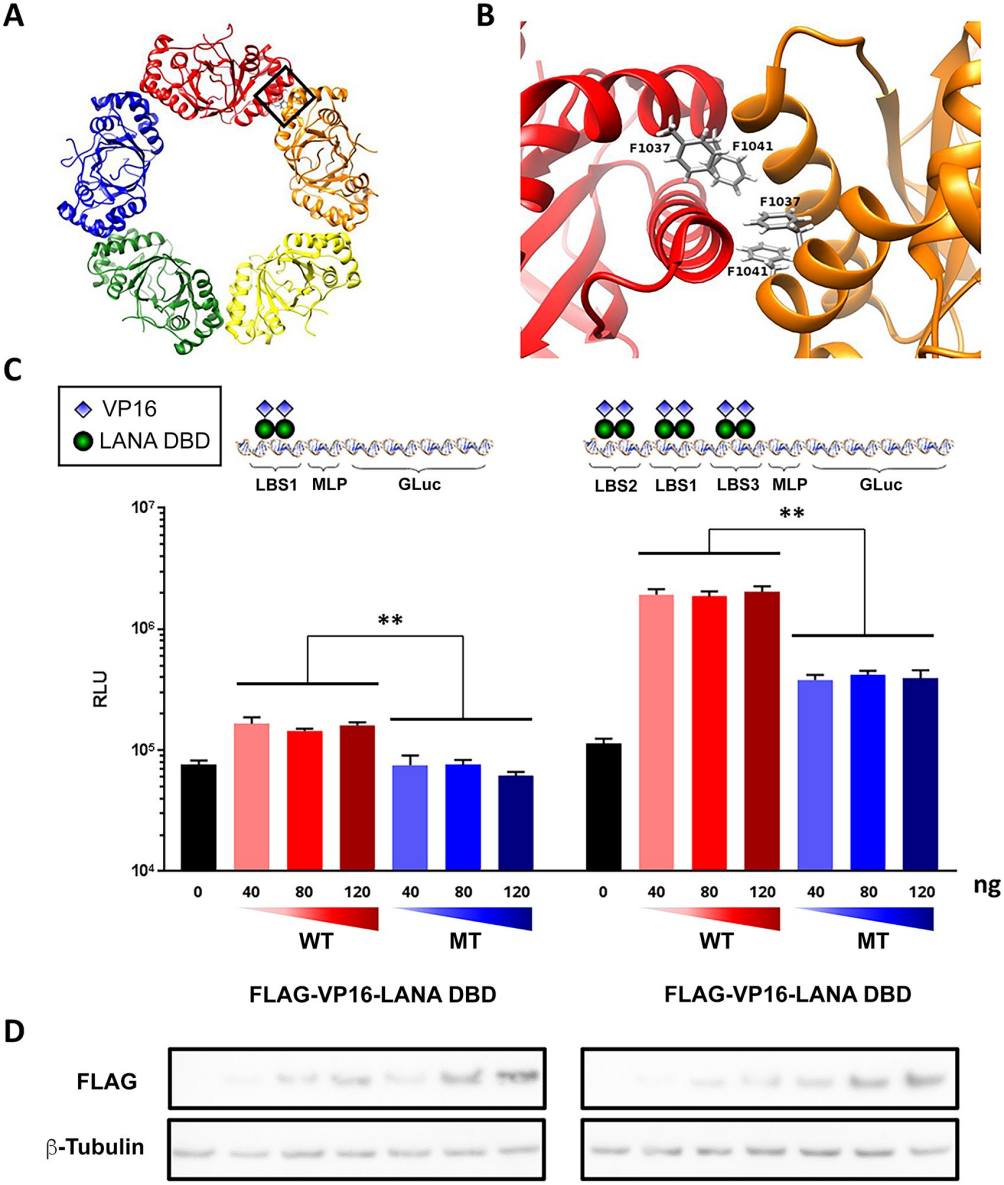
LANA oligomerization interface contributes to cooperative DNA binding at TR. Rendering of LANA crystal structure decamer **(A)** and oligomeric interface **(B)**. **(C)** Luciferase reporter with 1x (left panel) or 3x (right panel) LBS from TR was assayed in cells expressing FLAG-VP16-LANA DBD- WT or MT. ** p value < 0.01 using two-tailed student t-test. **(D)** The expression levels of FLAG-tagged VP16-LANA DBD- WT or MT as shown in **(C)** were assayed by Western blot with FLAG antibody, and β-tubulin control was shown below.

Previous structural and molecular genetic studies have demonstrated that amino acids F1037 and F1041 form the oligomerization interface of the LANA DBD (Fig 3A and 3B) [10]. Mutation of this oligomerization interface (F1037A/F1041A) had no effect on LANA DNA binding to a single LANA binding site (LBS), but were found to disrupt oligomerization and cooperative DNA binding *in vitro*, and inhibit LANA-dependent plasmid maintenance *in vivo* [10]. Now, we demonstrate that LANA oligomerization is necessary for functional binding to the TR *in vivo* (Fig 3C and 3D). To this end, LANA DBD wild type (WT) or LANA DBD containing oligomerization interface mutations (F1037A/F1041A) (MT) were fused to the VP16 transcriptional activation domain and FLAG-tagged. We then assayed these for their ability to bind and activate transcription from a luciferase reporter plasmid with either a single LANA binding site (1xLBS) or three naturally occurring LBS (3xLBS) as found in the TR origin of DNA replication (Fig 3C). We found that LANA DBD WT could activate transcription ~2 fold from 1xLBS and ~20 fold from 3xLBS reporter, while LANA DBD MT failed to activate 1xLBS and activated 3xLBS ~4 fold. This difference was more striking considering that LANA MT was expressed at higher levels than LANA WT, as detected by Western blot (Fig 3D). These findings indicate that LANA oligomerization interface contributes to the functional binding at the LBS in living cells and support the model that cooperative DNA binding is important for LANA function at the TR.

### LANA oligomerization interface is required for LANA nuclear body formation

**Fig 4:**
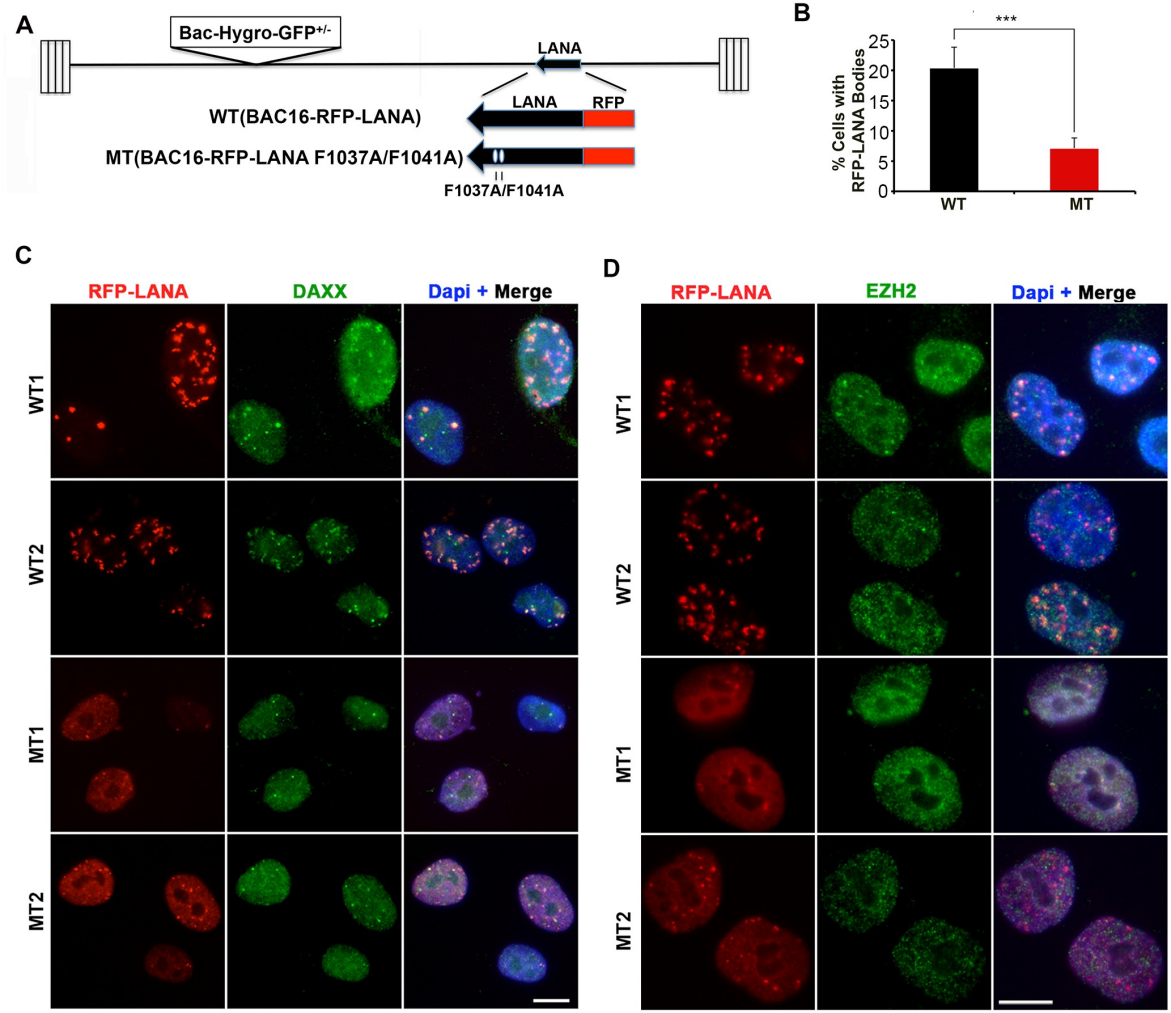
LANA oligomerization interface is required for LANA body formation and colocalization with DAXX and EZH2. **(A)** Schematic of KSHV Bac-RFP-LANA WT and Bac-RFP-LANA MT (F1037A/F1041A). **(B)** Quantification of RFP-LANA nuclear body formation in iSLK stable clones for WT and MT LANA. P-value was determined by two-tailed student t-test. **(C)** Indirect immunofluorescence analysis of the association of RFP-LANA (red) with DAXX (green) in RFP-LANA WT1 and WT2, or MT1 or MT2 stable iSLK cell lines. Cells were counter-stained with Dapi (blue) as shown in merge. Scale bar = 10 μm. **(D)** The same as in panel C, except imaging with EZH2 (green). Scale bar = 10 μm.

To investigate whether LANA oligomerization is required for the higher-order nuclear bodies observed by light microscopy, we engineered the F1037A and F1041A mutations into RFP-LANA BAC16 construct (Fig 4A). We generated at least two independent iSLK cell lines for each bacmid. We observed a significant difference in the frequency and abundance of nuclear bodies. LANA MT formed more diffuse nuclear fluorescence patterns compared to LANA WT (Figs 4B and S1A). LANA nuclear bodies were observed during primary infection of SLK (S1B Fig) and HUVEC (S1C Fig) cells. LANA WT formed visible foci in the nuclei of infected cells within 48 hrs post-infection. In contrast, LANA MT formed more diffuse and less intense fluorescence signals at the same time point (S1B and S1C Fig). In stable iSLK cells, RFP-LANA WT colocalized with DAXX and EZH2 in nuclear foci, while RFP-LANA MT appeared as diffuse pattern with dispersed colocalization (Fig 4C and 4D). LANA oligomerization mutation did not disrupt the ability of LANA to co-immunoprecipitate with DAXX (S2 Fig), consistent with other studies mapping a DAXX interaction interface to regions outside the LANA DBD [27]. These finding indicate that mutations in the LANA oligomerization interface (F1037A/F1041A) reduce the ability of LANA to form nuclear bodies that colocalize with DAXX and EZH2 in living cells (S3 Fig).

### LANA oligomerization mutants destabilize LANA binding and ORC recruitment to viral TR

**Fig 5:**
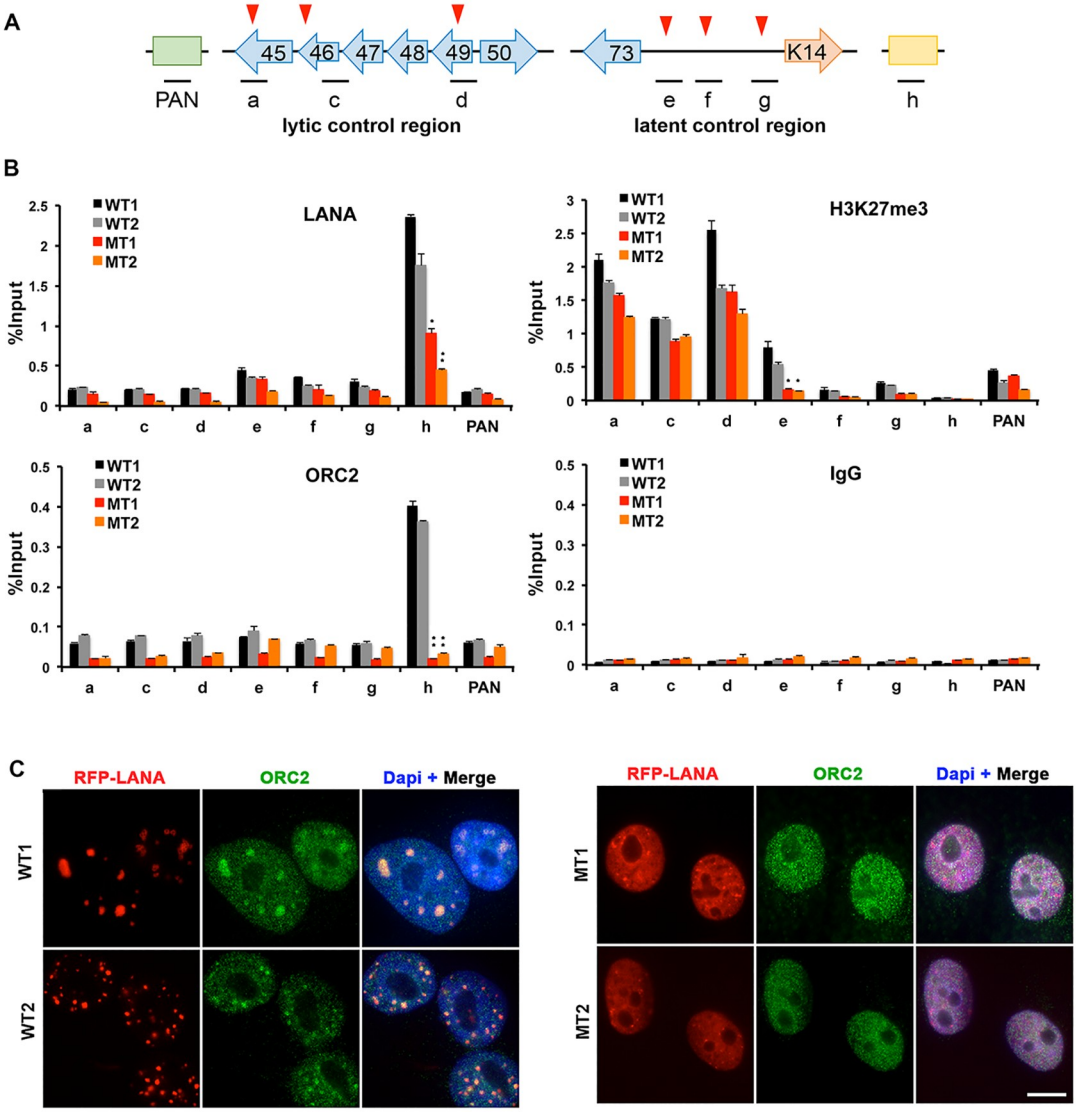
LANA oligomerization is important for LANA-binding and ORC recruitment to KSHV TR. **(A)** Schematic of ChIP-qPCR primer positions with relation to KSHV genes and loci. Red triangles indicate position of CTCF binding. **(B)** ChIP-qPCR analysis of LANA-RFP WT1, WT2, MT1, or MT2 stable iSLK cell lines using antibodies for LANA, ORC2, H3K27me3, or IgG control, as indicated. * p value < 0.05, ** p value < 0.01 using two-tailed student t-test. **(C)** RFP-LANA WT1 and WT2, or MT1 or MT2 stable iSLK cell lines were imaged with ORC2 (green) and Merge images were shown with Dapi (blue) staining. Scale bar = 10 μm.

To evaluate the potential effects of LANA oligomerization mutants on the KSHV epigenome, we assayed the interaction of several DNA-binding and chromatin regulatory factors with the KSHV episome by chromatin-immunoprecipitation (ChIP) assay (Fig 5). We first investigated LANA binding and observed that oligomerization mutants were significantly impaired for their ability to bind to the KSHV TR (primer h) relative to LANA WT (Fig 5A and 5B). This is consistent with the previous finding that mutant LANA is compromised for activating a TR-dependent reporter gene in vivo (Fig 3). ORC2, a factor known to be recruited to TR by LANA and functionally important for viral DNA replication and episome maintenance [16], was completely eliminated from TR in LANA MT relative to WT KSHV genomes (Fig 5B). Consistent with ChIP, we found that ORC2 colocalized with LANA WT foci, but only formed diffuse patterns with LANA MT (Figs 5C and S3). Histone H3K27me3 was found to be enriched at the lytic control region (regions a, c, d), but enrichment at this region was not significantly affected by LANA oligomerization mutants. On the other hand, intermediate levels of H3K27me3 found at the latency control region (primers e) were significantly reduced by LANA MT (Fig 5B). The pattern of binding of CTCF, H3K4me3, and total H3 were similar to that reported for BCBL1 [32], but this binding was not significantly affected by LANA oligomerization mutants (S4 Fig). RAD21 trended to be generally reduced throughout the KSHV genome in LANA MT relative to WT, although the levels of binding were relatively low throughout the genomes in iSLK cells (S4 Fig). These findings indicate that LANA oligomerization mutants have defects in binding to the TR, recruiting ORC2 to the TR, and maintaining H3K27me3 at the latency control region.

### LANA oligomerization mutants disrupt LANA auto-regulation and DNA loop formation between TR and the ORF73 control region

**Fig 6:**
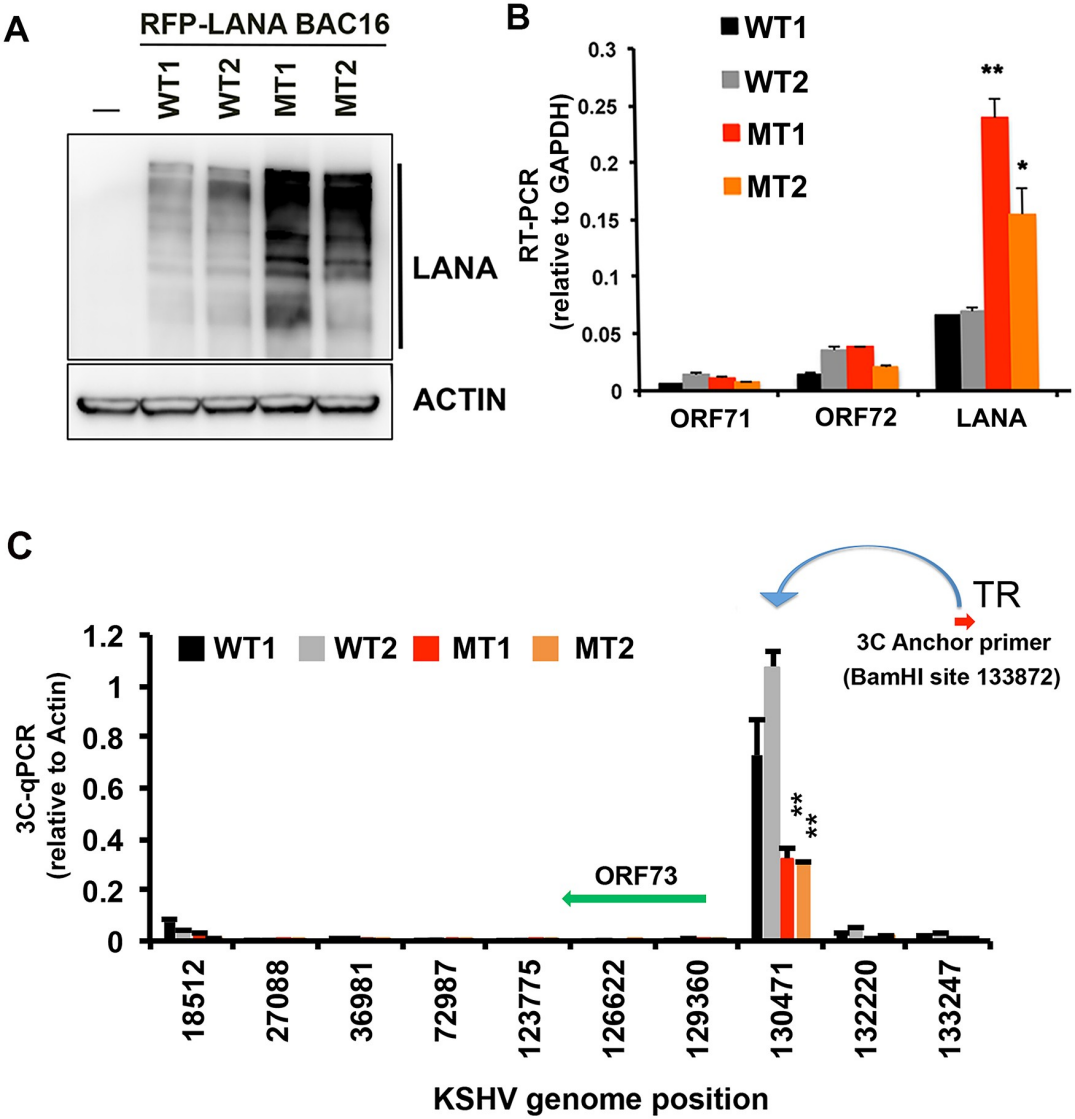
LANA oligomerization controls ORF73 auto-regulation and TR chromosome conformation. **(A)** RFP-LANA WT1, WT2, MT1, or MT2 stable iSLK cell lines were assayed by Western blot for LANA (top) and Actin loading control (lower). **(B)** RFP-LANA WT1, WT2, MT1, or MT2 stable iSLK cell lines were assayed by RT-PCR for expression of ORF71, ORF72, or LANA. mRNA was quantified relative to GAPDH. * p value <0.05, ** p value <0.01 using student t-test. **(C)** RFP-LANA WT1, WT2, MT1, or MT2 stable iSLK cell lines were assayed by 3C using anchor primer near TR (position 133872) and assayed at positions indicated on x-axis. 3C-qPCR relative to actin control is indicated. ** p value <0.01 using student t-test.

LANA oligomerization domain mutants were next assayed for LANA protein and RNA expression (Fig 6). iSLK cells carrying oligomerization domain mutants showed an increase in the total abundance of LANA protein, relative to cells carrying wildtype LANA for two independent lines of each WT or MT LANA (Fig 6A). On the other hand, LANA MT containing cells produced lower levels of viral lytic proteins ORF45 and ORF50 after doxycycline induction of RTA in iSLK cells (S5A Fig). These same cell lines were assayed by RT-qPCR for the expression of several viral genes including the latency transcripts for ORF71, ORF72, and ORF73 (Fig 6B), and lytic transcripts for ORF50, ORF45, and PAN (S5B Fig). Consistent with Western blot results, MT1 and MT2 produced higher levels of ORF73, but not ORF71 or ORF72 relative to WT controls (Fig 6B), and reduced levels of ORF45 transcript (S5B Fig). These findings suggest that LANA oligomerization mutants fail to negatively auto-regulate ORF73/LANA transcription.

To investigate potential mechanisms for LANA autoregulation, we used chromatin conformation capture (3C) to determine if the LANA binding at the TR may contact potential regulatory regions controlling the LANA transcript. We have previously observed a strong 3C DNA loop between the latency control and lytic control regions that was dependent on CTCF and cohesin subunit RAD21 in BCBL1 cells [33]. We found that this 3C interaction was weak in iSLK cells, but completely undetectable in genomes with LANA oligomerization mutants (S6 Fig). To determine if there were 3C interaction with the TR element and the LANA promoter, we positioned a 3C anchor primer adjacent to the BamHI site closest to the KSHV TR (Fig 6C). We then assayed whether this anchor primer could form any detectable loops with other regions of the KSHV genome. We found that the TR anchor formed a robust interaction with the region at 130471, which is situated ~3 kb upstream from the ORF73 transcription start site (Fig 6C). Importantly, this TR-mediated interaction was significantly reduced by LANA oligomerization mutants MT1 or MT2 relative to LANA WT. These findings suggest that LANA oligomerization is important for a viral chromosome conformation that involves contacts between the TR and other regulatory regions.

### LANA oligomerization is required for viral genome integrity

**Fig 7:**
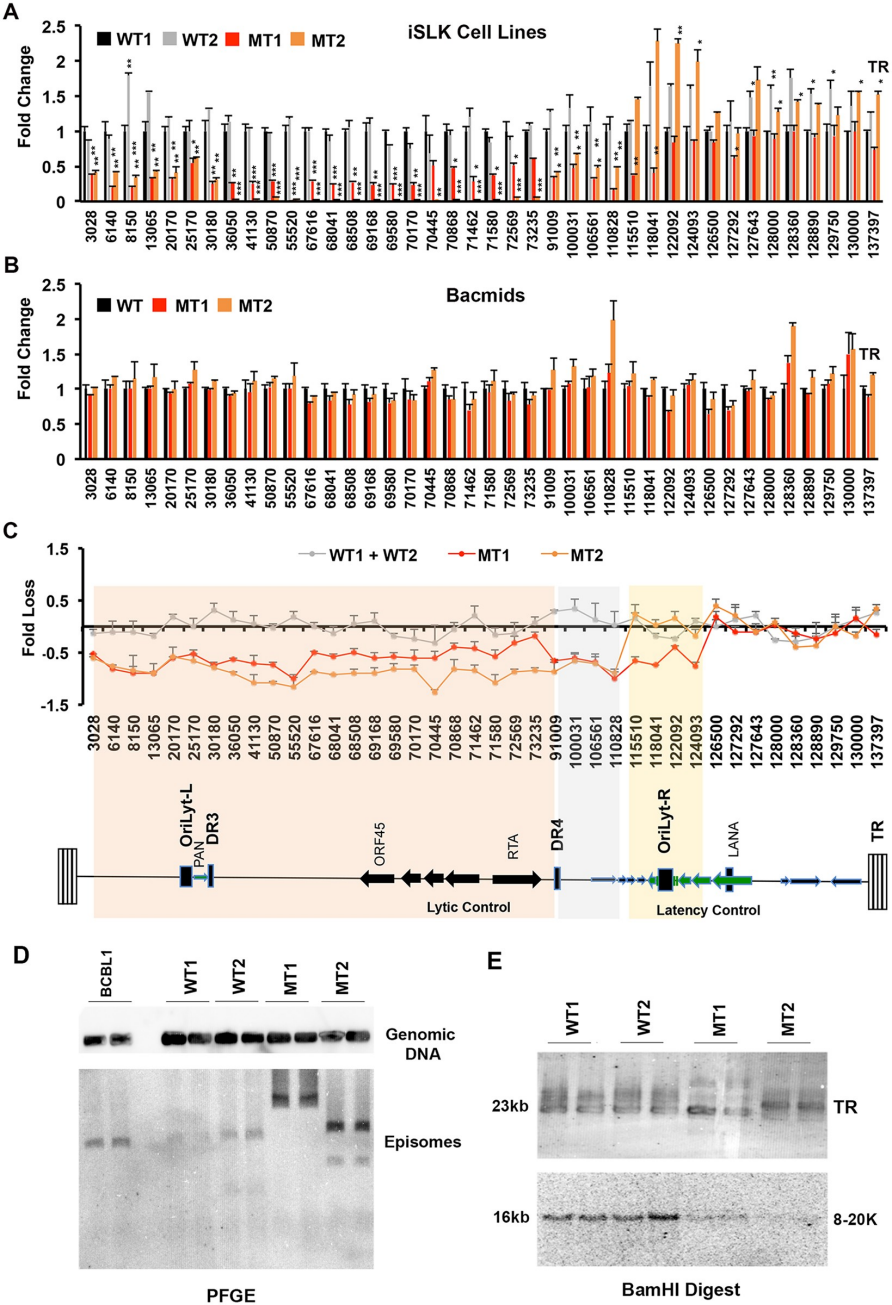
LANA oligomerization is important for viral genome integrity. **(A)** RFP-LANA WT1, WT2, MT1, and MT2 stable iSLK cell lines were analyzed by qPCR for copy number variation using primers spanning KSHV genome, as indicated on X-axis. **(B)** RFP-LANA WT, MT1, and MT2 bacmid DNA was quantified by qPCR as in panel A. **(C)** Fold loss of KSHV DNA relative to bacmid control for MT1, MT2, and the average of WT1+WT2. Region of genetic instability are highlighted with salient viral genes and features indicated. * p value < .05, ** p value < .01 using student t-test. **(D)** BCBL1 or KSHV Bac RFP-LANA WT1, WT2, MT1, or MT2 were analyzed by agarose plug PFGE and Southern blot with KSHV bacmid probe (Episomes) or 18S DNA (Genomic DNA). Agarose plugs containing 10^6^ cells were analyzed in duplicate. **(E)** Total genomic DNA from or KSHV Bac RFP-LANA WT1, WT2, MT1, or MT2 was digested with BamHI and assayed by PFGE and Southern blot with probes to TR (top) or KSHV genomic DNA between coordinates 2kb-18kb on the KSHV genome.

Since LANA oligomerization mutants have defects in long-term episome maintenance, we considered the possibility that mutant viral genomes may be genetically unstable. We therefore quantified by qPCR a series of viral genomic regions in total DNA isolated from iSLK clones carrying MT and WT LANA (Fig 7). We identified a large region spanning from base pair 1 to 110,000 in the virus genome that had a significantly reduced copy number relative to bacmid hygromycin gene sequence (Fig 7A and 7C). This region contains lytic control genes and duplicated lytic origins. We did not detect any consistent loss of copy number at the latency control region and TR proximal regions ranging from 126.5–137.3 kb of the viral genome. Importantly, we did not detect any copy number variation in the starting bacmid DNA for MT1 or MT2 relative to the WT bacmid used for generating iSLK stable cell lines (Fig 7B). Sequencing of these starting bacmids did not reveal any deletions or mutations other than the expected point mutations in the LANA DBD (S1 Data). PFGE analysis of these cells indicated that episomal genomes were maintained in all cell lines, but gross rearrangements relative to parental and WT genomes were observed with MT1 and MT2 (Fig 7D and 7E). Similar genetic instability and viral phenotypes were observed in an independently derived pair of cell lines using a different pair of bacmid clones (WTgfp and MTgfp), indicating that these phenotypes are attributable to LANA oligomerization mutation followed by propagation in cell culture (S7–S9 Figs). These findings suggest that LANA oligomerization mutants fail to maintain the integrity of the complete KSHV genome, with loss of DNA encompassing the lytic origins and lytic control regions after prolonged selection in cell culture.

## Discussion

**Fig 8:**
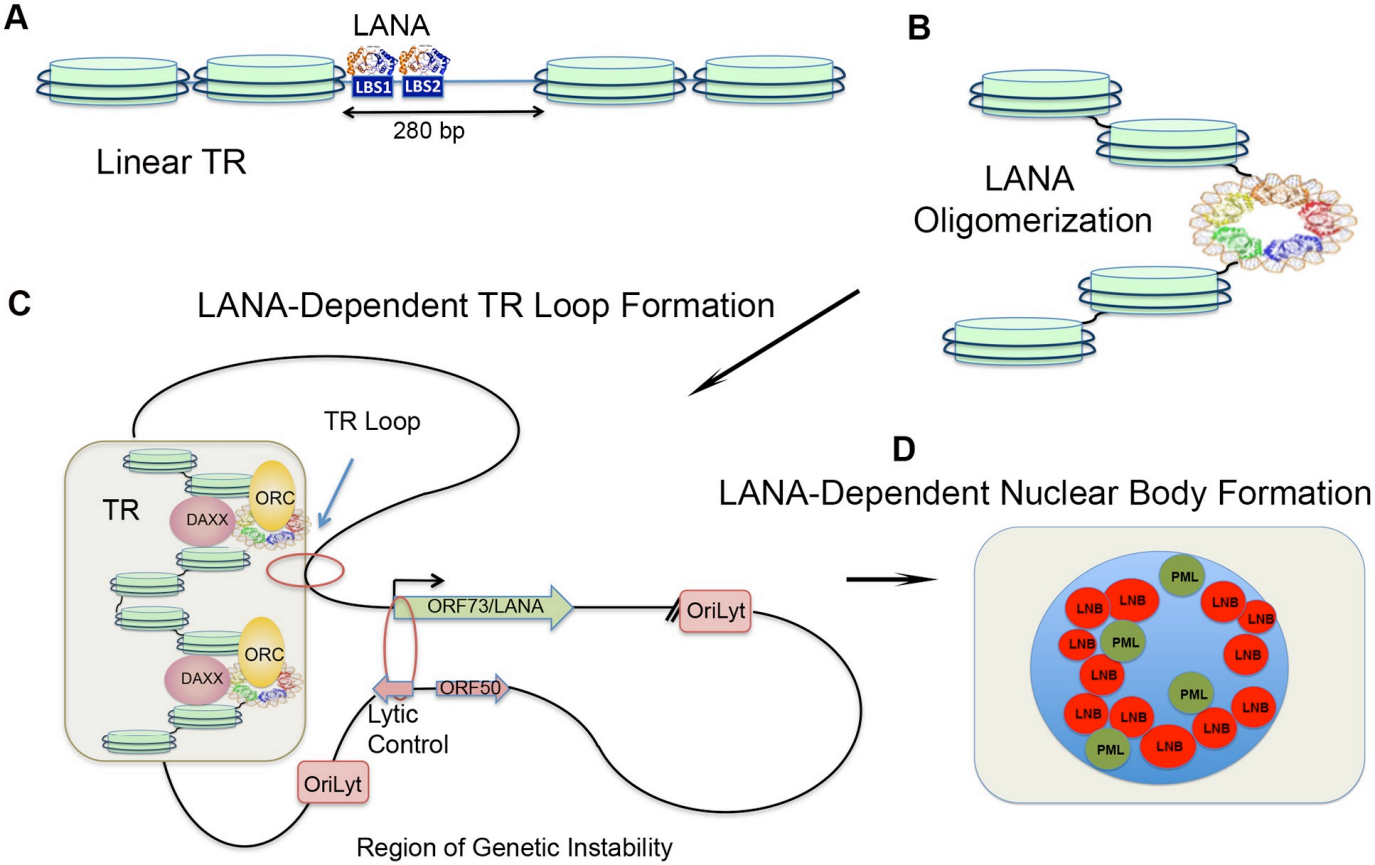
Model of LANA oligomerization induced changes in KSHV TR conformation and function. (A) Depiction of TR bound to non-interacting, low affinity monomeric LANA or, (B) LANA oligomerization-induced conformational change in TR in the context of nucleosomes. Histone is shown in turquoise. (C) LANA oligomerization-induced conformation change in TR recruits additional factors, such as Daxx and ORC, and enables KSHV genome DNA loop interactions between TR and the LANA transcriptional regulatory region. LANA oligomerization also regulates the transcription and replication control necessary to maintain viral genome stability. Regions of viral genetic instability is indicated from OriLyt to TR. **(D)** LANA oligomerization is also required for the formation of LANA nuclear bodies (LNB) (red), distinct from PML-NBs (green), that can be transmitted as stable structures throughout mitosis.

Formation of complex, higher-ordered structures from simple repetitive units is a common theme in biological systems [34]. LANA is a multifunctional protein that binds cooperatively to three tandem recognition sites in a single viral TR. The viral TR is an ~850 bp element that is tandemly repeated as many as 35 times in some viral genomes. Multiple tandem copies of TR are required for LANA-dependent long-term stable episome maintenance of the complete viral genome [15, 35]. How LANA builds higher-ordered functional structures from this repetitive binding block is not completely clear. Here, we provide evidence that the simple homo-oligomeric interactions of the LANA DBD drive formation of higher-order chromatin architecture and nuclear domain structures required for stable episome maintenance of KSHV genomes during persistent latent infection (Fig 8).

LANA can self-assemble as a variety of different oligomeric forms in X-ray crystallographic lattices, including decamers, pentamers, and spirals [9–12]. LANA can also form continuous filamentous oligomers when bound to non-specific DNA in electron microscopy studies [9]. There is evidence that LANA can initiate additional DNA interactions through a basic patch on the back surface of the sequence-specific DNA binding domain [9]. LANA is also known to have an N-terminal nucleosome interaction domain important for tethering to metaphase chromosomes and essential for episome maintenance [20]. These multiple DNA and chromatin interactions by LANA contribute to its ability to form complex architectural structures. Previous studies have shown that mutations in the LANA oligomerization interface have no measurable effect on LANA-DNA binding to a single LBS, but did have defects in cooperative binding to larger DNA containing multiple LBS as found in native TR elements [10]. We now find that LANA oligomerization mutants are compromised for binding to KSHV TR in living cells. This suggests that oligomerization is required for stable binding to multiple LBS in the TR. LANA oligomerization and cooperative binding may be necessary to overcome the energetic barriers of DNA and nucleosome structures that prohibit monomeric LANA binding. Our data suggest that cooperative assembly and precise geometry of LANA oligomers at TR is critical for the formation of these higher order structures and their associated functions.

The KSHV genome can form complex DNA interactions, including a DNA loop between the latent and lytic control regions that are mediated by CTCF-cohesin interactions [33, 36]. We now report the identification of an additional 3C DNA loop between the TR and the region upstream of the LANA promoter. Supporting evidence for this interaction is provided by ChIP-Seq experiments showing a high affinity interaction with the TR and a secondary, lower affinity LANA interaction with the region upstream of LANA transcription start site [37, 38]. The TR anchored DNA loop was disrupted by mutations in the LANA oligomerization interface, suggesting that higher-order LANA oligomerization is required for loop formation. Since LANA oligomerization was also required for efficient TR binding as well as ORC2 and RAD21 recruitment, it is likely that these factors and others also contribute to the formation of a stable DNA conformation. The function of this LANA-dependent loop may be in the auto-regulation of the LANA transcript. We observed that the LANA/ORF73 transcript and protein were up-regulated in the presence of mutations that abrogate LANA oligomerization. We suspect that LANA DNA loop formation between TR and LANA transcriptional regulatory region plays a role in the auto-repression of LANA transcription. Similar autoregulation is observed for EBNA1 binding to its transcription initiation site at the Qp promoter in the EBV genome [39, 40]. Transcriptional autoregulation has been implicated in the copy number control mechanisms for several plasmid systems, including the yeast 2 micron plasmid [41], and may play an important role in regulating KSHV episome copy number and latency control.

Genomes containing mutant LANA failed to maintain genetic stability over time, with a loss of viral DNA from regions more distal to the TR and latency control region. One potential explanation for this loss of genomic DNA is that TR fails to recruit ORC and function efficiently as a replication origin. Inefficient origin firing could result in the incomplete replication of the viral genome over multiple cell divisions. We also observed that the boundary for genetic loss appears to coincide with the TR and the right lytic origin (KSHV genome coordinates ~1–110,000), suggesting that lytic origin activation may have destabilized this region of the genome. Although the mechanism for loss of genetic material is not completely understood, these findings underscore the crucial role of LANA oligomerization in maintaining viral genome integrity.

The super-molecular structures formed by LANA and KSHV episomes can be readily visualized by light microscopy as LANA nuclear bodies. Large LANA bodies were observed in a large percentage of cells carrying LANA WT, but to a much lesser extent in oligomerization defective LANA MT. This suggests that LANA oligomerization is the driving force for the formation of these higher order structures. These large structures form at much higher frequency in cells where KSHV episomes are stably maintained, suggesting that additional epigenetic or stochastic factors contribute to the formation of a stable LANA nuclear body. We observed a high colocalization of LANA nuclear bodies with DAXX. These bodies lacked PML, suggesting they are distinct from traditional PML-NBs. DAXX has been implicated in histone H3.3 chaperone activity and transcription repression [42–44], but may also play a role in macromolecular protein complex assembly [45]. We suggest that the function of DAXX in these LANA nuclear bodies is to facilitate formation and stabilize these large multi-protein, nucleic acid complexes. We also observed partial colocalization with EZH2 and H3K27me3, consistent with other studies showing the importance of polycomb repression in maintaining KSHV latency [28, 46–48]. We also observed that ORC2 colocalized with a large fraction of LANA bodies. Numerous other factors have been shown to colocalize with LANA, including γH2AX [49], H3K9me3[50], KDM3A[51], RFC[52], NRF2 [53], as well as DAXX [26, 27, 54]. Whether these other factors contribute to the formation of LANA nuclear bodies is not known. LANA nuclear bodies are typically only observed when LANA is associated with viral genomes [15]. Super-resolution microscopy of LANA interaction with 2xTR DNA revealed LANA can nucleate a hub at the TR with multiple interactions to host chromatin and bending of nucleosomal DNA consistent with activated chromatin [29]. Live cell imaging using fluorescently tagged episomes indicate that KSHV genomes form multicopy clusters, that tend to segregate to a single daughter cells [30]. Our live cell imaging of RFP-LANA in stable iSLK cells suggests that the LANA nuclear bodies form large clusters and is consistent with the observation that these clusters can interact dynamically. However, we observed that LANA nuclear bodies are transmitted faithfully to daughter cells and retain similar copy number and perinuclear distribution as observed in the parent cell. We propose that these various and heterogeneous super-molecular LANA bodies acquire an epigenetic memory allowing their stable transmission through mitotic cell division.

Super-molecular structures are observed in many complex biological processes, including transcription, replication, and viral assembly. Chemical phase-changes due to the cooperative interactions of multi-valent proteins are thought to be the driving force for the assembly of these super-molecular structures [34, 55]. Our findings support the model that modest changes in the molecular geometry of KSHV LANA can have a profound effect on the macro-molecular structure and biological function of the KSHV episome, including phenotypes in transcription, replication, and super-structures important for genome integrity and transmission (Fig 8). We propose that stable protein oligomeric architecture drives higher-order structures important for viral chromosome architecture and episome maintenance.

## Materials and methods

### Cell lines and culture conditions

iSLK (gift for Don Ganem, Novartis) cells were cultured in Dulbecco’s modified Eagle’s medium (DMEM) supplemented with 10% fetal bovine serum (heat inactivated) and 1% penicillin-streptomycin in the presence of 1μg/ml puromycin, 250 μg/ml G418. SLK (gift of Don Ganem) and BCBL1 cells (gift of Yan Yuan) were cultured in RPMI-1640 medium supplemented with 10% FBS and 1% P/S. iSLK RFP-LANA and iSLK GFP-RFP-LANA cells were cultured in iSLK growth medium with additional 1200 μg/ml hygromycin B. HUVEC cells (ATCC PCS-100-010) grown in complete Endothelial Cell Growth Medium (CELL applications, 211–500).

### BAC mutagenesis and plasmids

The KSHV bacmid BAC16 was kindly provided by Dr. Jae U. Jung and K. Brulois (University of Southern California). The KSHV BAC16 clone was modified by a two-step Red-mediated mutagenesis as described previously [56]. RFP was fused in frame to the N-terminus of LANA using the pEPmRFP-in plasmid as template for PCR amplification with primers oPL6253 and oPL6254 (S1 Table). A founder RFP-LANA bacmid clone was isolated and validated by restriction digest and sequencing of the entire genome. RFP-LANA bacmids were further confirmed for RFP-LANA expression and production of infectious virus. We next removed the GFP gene from the bacmid backbone of the RFP-LANA bacmid. GFP was deleted using the primers oPL7003 and oPL7004 (S1 Table). The RFP-LANA bacmids (with and without GFP) were then used to generate the LANA oligomerization domain mutant F1037A/F1041A using the primers oPL6189 and oPL6190 (S1 Table). Mutations were validated by sequencing and restriction digest to confirm integrity of the viral genome. Two clones MT1 and MT2 were further characterized.

### BAC sequencing

Sequencing was done on the NextSeq 500 platform using medium output run with 75bp single end. Samples bowtie2 [57] was used to align samples against the reference allowing for 2 mismatches and up to 200 multiple maps. Samtools [58] algorithm was then used call mutations and indels. Every sample was also quantified for each reference position to count number of A/T/C/G reads that had quality of at least 15 using bam-readcount tool. The sequence of Bac16 RFP-LANA WT1, WT2, MT1 and MT2, not including the BAC insertion, has been submitted to GenBank (accession no. MK143395) (S1 Data).

### BAC16 iSLK cells

BAC16 and its derivatives were transfected into iSLK cells using the Effectene Transfection Reagent kit (QIAGEN, 301425). Briefly, BAC16 DNA was isolated from 2ml bacterial culture using a ZR BAC DNA Miniprep Kit (Zymo Research) and resuspended in 30 μl of RNase-free water. iSLK cells were seeded at 2x10^5^ cells/well of a 6-well plate. On the next day 15 μl of BAC DNA was mixed with transfection solution and added to the cells following by transfection kit’s according to manufacturer’s protocol. On following day, transfected cells were trypsinized and transferred into 10 cm dishes and cultured in DMEM supplemented with 10% FBS and 1% penicillin-streptomycin. Two days later 1 μg/ml puromycin, 250 μg/ml G418 and 1,200 μg/ml hygromycin B were added to the growth media.

### Production of BAC16 virus stock

For virus stock preparation the BAC16 stable iSLK cells were treated with 1mM sodium butyrate and 1 μg/ml Doxycycline in the absence of hygromycin B, puromycin and G418. 4–5 days later, supernatant was collected, centrifuged (1,500 rpm for 10 min at 4°C) and filtered (0.45 μm). Virus particles were pelleted trough 25% sucrose by ultracentrifugation using 27,000 rpm for 1 hour at 4°C. The virus pellets were resuspended in RPMI-1640 and prepared to titer the virus and future infection.

### Indirect immunofluorescence (IF) assay

1x10^5^ (~80% confluence) cells were plated on 1cm round glass coverslips in 24-well-plate. Next day the cells were washed with PBS and fixed for 15 min with freshly prepared 2% paraformaldehyde in PBS, washed twice with PBS and permeabilized with 0.3% TX-100. After washing with PBS, the cells were incubated in blocking solution (0.2% fish gelatin, 0.5% BSA in PBS) for 30 min at room temperature. Primary antibody was diluted in blocking solution and applied to the coverslips for 1h followed by 3 times wash with PBS. The cells were further incubated with fluorescence-conjugated secondary antibodies for 1hr, counterstained with Dapi, and mounted in Fluoromount G medium (SouthernBiotech). Images were taken at Nikon Upright Microscope using 100X objective and processed by Adobe Photoshop CS5. Bleed-through control for RFP-LANA was performed for all IF experiments, in which coverslips were stained only with secondary antibody in the absence of primary antibody staining. We did not observe any bleed-through signals from RFP-LANA. For the α-tubulin staining of mitotic cells, iSLK RFP-LANA WT cells were seeded into 24-well plate with glass coverslips. After 72 h of growing the cells were washed with PBS, after fixed with cold 100% methanol, washed with PBS and stained with anti–α-Tubulin conjugated with AlexaFluor488 and Dapi in the end, and mounted in Fluoromount-G medium. Colocalization was determined and quantified using Nikon NIS Elements AR software, version 5.02 using the Spot Detection Tool to threshold foci for RFP-LANA and either Daxx, EZH2, or ORC2 based on fluoresecence intensity, object size(diameter) and object shape. Binary masks were generated for each pair and the two binary layers are then combined to create a third binary layer using a having operation that identifies spatial overlap of object pixels. The binary masks were used to calculate the number of RFP-LANA overlaps with either Daxx, EZH2, or ORC2.

### Confocal fixed and live cell imaging

High resolution, confocal images of mitotic cells in both fixed and live-cell configurations were captured using a Leica TCS SP8 WLL scanning laser confocal microscope with resonant scanner and Leica LAS-X software (Leica Microsystems, Inc., Buffalo Grove, IL), using imaging parameters which were chosen to both reduce photobleaching as well as minimize phototoxic effects. Image post-processing included importing into Huygens software for deconvolution (Scientific Volume Imaging, Laapersveld, Hilversum, The Netherlands) followed by maximum projection or 3D reconstruction, iso-surface application and video rendition in LAS-X.

Fixed cell preparations were acquired according to Nyquist parameters using a 63X/1.40 oil objective, 6X zoom and a pinhole of 0.48 AU, and 34 z-steps through 6.5 um stacks, resulting in a voxel size of 51 x 51 x 200 nm. Cells were labeled with DAPI (nuclei), Alexa488 (α–Tubulin) and RFP (LANA) and acquired with HyD detectors in sequence to maximize signal and minimize cross-talk. 6 line accumulations allowed laser intensities to be kept to 0.5% at 405 nm, 0.5% at 488 nm and 2% at 557 nm.

Original time-lapse sequences were acquired with cells incubated in 35 mm MatTek dishes (MatTek Corp., Ashland, MA) and maintained in a Tokai-Hit stage-top incubation chamber (Tokai Hit, Shizuoka-ken, Japan) at 37 °C and 5% CO2. Imaging hardware parameters were adjusted to preserve cell viability, including laser intensity settings of 0.3% at 488 nm for GFP and 2.0% at 555 nm for RFP, 4 line accumulations and use of HyD detectors. Stacks of images were captured with a 40X/1.30 Oil objective and 2X zoom at 10 locations over 48 hrs with a 10 min sampling interval, totaling 289 time points at each location. Each stack was 26 um thick and comprised of 34 sections in 0.8 um steps.

### Antibodies

The following antibodies were used for immunofluorescence studies: rabbit anti-Daxx (Sigma, D7810), rabbit anti-ATRX H-300 (Santa Cruz, Sc15408), rabbit anti-PML (Bethyl, A301167A), rabbit anti-EZH2 (Cell Signaling, 4905S), rabbit anti-H3K27me3 (Active motif, 39155), mouse anti-ORC2 (MBL, M0553), and mouse anti-αTubulin/Alexa488 (Invitrogen, 322588). Secondary antibodies AlexaFluor488 or AlexaFluor594 were purchased from Invitrogen. The following antibodies were used for Western blotting: mouse anti-ORF50 (provided by Erle Robertson, UPENN), mouse anti-ORF45 (provided by Yan Yuan, UPENN), rat anti-LANA (Advanced Biotechnologies Inc., 13210), rabbit anti-Daxx (Sigma), and anti-actin-HRP (Sigma, A23852). Antibodies used in ChIP assay include: rabbit polyclonal antibodies to histone H3K4me3 (Millipore, 07473), histone H3K27me3 (Active motif,391155), total histone H3 (Bethyl), ORC2 (MBL, M0553), Rad21 (Abcam, ab992), CTCF (Millipore, 07729), or rabbit IgG (Santa Cruz Biotechnology, sc-2027), and rat polyclonal anti-LANA (Advanced Biotechnologies Inc.).

### Western blot analysis

Equal amounts of protein extract in RIPA buffer (50mM Tris-HCl (pH8.0); 150mM NaCl; 1% NP-40; 0.5% Sodium deoxycholate; 0.1% SDS; 1mM EDTA) were resolved in 8–16% Novex Tris-Glycine gels (Invitrogen), and then transferred onto a PVDF membrane (Millipore), where they were incubated with specific antibodies followed by HRP-conjugated secondary antibodies (BioRad) and ECL reagents (Millipore) for detection.

### Co-Immunoprecipitation (Co-IP)

RFP-LANA WT and MT iSLK cells were washed 2 times with cold PBS, and then lysed in cold lysis buffer (20 mM Tris, pH 8.0, 137 mM KCl, 1 mM EDTA, 1.5 mM MgCl_2_, 10% Glycerol, and 1% Triton X-100 supplemented with 1 mM DTT and 0.1% mammalian protease inhibitor cocktail mix) for 30 mins on ice. Cell lysates were centrifuged at 13000 rpm for 10 mins, and the supernatants were precleared with Protein G Sepharose beads (GE Healthcare) for 60 mins at 4 °C with rotation. One ml of precleared lysates (~5 x 10^6^ cells) were immunoprecipitated with either rat anti-LANA (Advanced Biotechnologies Inc.) or mouse monoclonal anti-p53 (CalBiochem) or rabbit anti-DAXX (Sigma) overnight at 4 °C with rotation. The immuno-complex was collected with Protein G sepharose beads with rotating at 4 °C for 3 hrs, and the beads were washed 3 times with BC300 (300 mM KCl, 20 mM Tris-HCl, pH 8.0, 0.2 mM EDTA, 10% glycerol, and 10 mM β-mercaptoethanol) followed by once with BC100 at 4 °C. Pulled down proteins were eluted by boiling with 2x Laemmli buffer (100 mM Tris-HCl, pH 6.8, 4% SDS, 0.2% Bromophenol Blue, and 20% Glycerol), and were subject to SDS-PAGE and Western blot analysis.

### Quantification of viral intracellular DNA and Bacmids DNA

The amount of intracellular KSHV DNA and bacmid DNA were determined by quantitative PCR (qPCR) analysis using primers specific for KSHV genome (listed in S2 Table). The data were normalized to the hygromycin DNA region, as described previously [32].

### *Gaussia* Luciferase Assay

HEK293T cells were seeded at a density of ~200,000 cells/well in 12-well plates. The following day, the cells were transfected with a *Gaussia* luciferase reporter plasmid (containing a single LBS1 or LBS2-LBS1-LBS3) and a plasmid for expression of a fusion protein containing an N-terminal FLAG tag, the VP16 (activation domain), and the wild-type (wt) or mutant (F1037A, F1041A) LANA DNA-binding domain (DBD). An empty FLAG vector was used to ensure that the total amount of DNA per transfection was equal to 225 ng. Transfections were performed using Lipofectamine 2000 at a ratio of 1:3 (mass of DNA per volume of transfection reagent) in antibiotic-free media. After 6 hours, the antibiotic-free media was replaced with media containing antibiotics. Approximately 18.5 hours later, luciferase measurements were made by transferring 40 μL of media and 10 μL of substrate (BioLux Gaussia Luciferase Assay Kit, New England Biolabs) to a 96-well plate and measuring luminescense with an Envision plate reader (PerkinElmer). Cells were lysed with RIPA buffer containing 1 mM PMSF and supernatants were run on a NuPAGE 4–12% Bis-Tris Gel (Invitrogen) and transferred to a Power Blotter nitrocellulose membrane (Invitrogen) using a Power Blotter (Invitrogen). The membrane was probed using anti-FLAG M2-Peroxidase antibody (1:10,000) (Sigma-Aldrich) or β-tubulin loading control antibody (1:4,000) (Fisher).

### Chromatin immunoprecipitation (ChIP) assay

ChIP assays were performed as described previously [59]. Briefly, cells (~ 1 x 10^7^) were crosslinked in 1% formaldehyde with shaking for 15 min, quenched by the addition of glycine to a final concentration of 0.125 M, and lysed in 1 ml SDS lysis buffer (1% SDS, 10 mM EDTA, and 50 mM Tris-HCl, pH 8.0) supplemented with 1 mM PMSF and protease inhibitor cocktails (Sigma-Aldrich). The lysates were sonicated with a Diagenode Bioruptor, cleared by centrifugation to remove insoluble materials, and diluted 10 fold into IP Buffer (0.01% SDS, 1.1% Triton X-100, 1.2mM EDTA, 16.7mM Tris pH 8.1, 167mM NaCl, 1 mM PMSF, and protease inhibitors cocktail) for IP reaction at 4°C overnight. Each immune complex was washed five times (1 ml wash, 10 mins each) in ChIP related wash buffer at 4°C, eluted by addition of 150 μl Elution buffer (10mM Tris, pH 8.0, 5mM EDTA, and 1% SDS) at 65°C for 30 min, and the elutes were placed at 65°C for overnight to reverse cross-linking. The elutes was further treated with Proteinase K in a final concentration of 100 μg/ml at 50°C for 2 hrs, and ChIP DNA was purified by Quick PCR Purification Kit (Life Technologies) following the manufacturer’s instruction. ChIP DNA was assayed by qPCR using primers specific for indicated KSHV regions and quantified as % input.

### Chromatin conformation capture (3C)

3C experiments were carried out as described previously [33], with minor modifications. PCR data were normalized to cellular actin. Briefly, 5×10^6^ KSHV iSLK WT or MT cells were cross-linked with 1% formaldehyde at room temperature for 10 min, followed by the addition of glycine at the final concentration of 125 mM for 10 min at room temperature. Cellular pellets were washed once with cold PBS and resuspended in 250 μL of ice cold lysis buffer (10 mM Tris-HCl, pH 8; 10 mM NaCl; 0.2% Igepal CA630;1 × complete protease inhibitor; 11836145001 Roche) for 30 min with rotation at 4°C, centrifuged for 5 min at 2500 g at 4°C and then washed once with 500 μL of ice-cold lysis buffer. Resultant pellets were gently resuspended in 100 μL of 0.5% SDS and incubated at 65°C for 10 min. After addition of 50 μL of 10% Triton X-100 to quench the SDS and 290 μL of distilled water, samples were incubated at 37°C for 15 min, centrifuged for 5 min at 2500 g at 4°C and washed once with 500 μL of ice-cold lysis buffer. Pellets were resuspended in 200 μL of 1X restriction enzyme buffer and digested with 100 units of BamHI overnight at 37°C. To inactivate the restriction enzyme, the digested samples were incubated at 65°C for 20 min. The samples were ligated in 1 mL of ligation buffer (1X T4 ligase buffer, 1% Triton X-100, 8μg/mL BSA, 10000 CEU of T4 DNA ligase) at room temperature for 6 hours with slow rotation. Ligated samples were centrifuged for 5 min at 2500 g at 4°C, resuspended in 368 μL of lysis buffer and treated with 10 μl of 20 mg/ml Proteinase K, 20 μL of 10% SDS, 40 μL 5M sodium chloride at 65°C overnight to reverse cross-linking. Genomic DNA was extracted with phenol/chloroform, resuspended with 250 μl of distilled water and subjected to qPCR using primers listed in S3 Table.

## Supporting information

S1 Fig LANA oligomerization domain contributes to LANA nuclear body formation. **(A)** Immunofluorescence analysis of RFP-LANA foci in RFP-LANA WT (left) or MT (right) iSLK stable cells. Representative images showed RFP-LANA (red) and Dapi staining (blue). Scale bar = 10 μm. **(B-C)** SLK **(B)** or HUVEC **(C)** cells were infected with RFP-LANA WT or MT KSHV bacmid virus and immunofluorescence analysis was used to assay RFP-LANA WT (top) and MT (bottom) at 48 hrs post primary infection. Infected cells were shown by GFP (green) and counter-stained by Dapi in merged images. Scale bar = 10 μm.
(TIF)

S2 Fig LANA oligomerization is not required for interaction with DAXX. RFP-LANA WT (left) or MT (right) expressed from BAC16 in stable iSLK cells was subject to IP with either IgG, LANA, p53, or DAXX antibodies, and then assayed by Western blot with antibody to LANA (top), DAXX (middle), or p53 (lower).
(TIF)

S3 Fig Quantification of colocalization by IF imaging. **A)** The percentage of foci for ORC2, DAXX, or EZH2 that colocalized with LANA bodies for RFP-LANA WT (black) and RFP-LANA MT (red). **B)** The percentage of cells in the population that display colocalized nuclear structure for ORC2, DAXX, or EZH2 in RFP-LANA WT (black) and RFP-LANA MT (red). Colocalization was determined and quantified using Nikon NIS Elements AR software, version 5.02 using the Spot Detection Tool. **p value < .01, *** p value <0.001 was calculated using two-tailed student t-test. **(C)** Example of computational method for quantifying colocalization of LANA and DAXX foci. The colored circular outlines indicate the number of Daxx (green) and RFP-LANA (red) foci. The white outlines in the merged image show the number of LANA foci colocalized with Daxx foci. Bar scale = 10um.
(TIF)

S4 Fig LANA oligomerization does not affect CTCF, H3K4me3, RAD21 binding to KSHV genome. **(A)** Schematic of ChIP-qPCR primer positions with relation to KSHV genes and loci. Red triangles indicate position of CTCF binding. **(B)** ChIP-qPCR for LANA-RFP WT1, WT2, MT1, or MT2 stable iSLK cell lines using antibodies for CTCF, H3K4me3, RAD21, and histone H3 as indicated.
(TIF)

S5 Fig LANA oligomerization mutants are compromised for lytic reactivation. **(A)** RFP-LANA WT1, WT2, MT1, or MT2 stable iSLK cell lines were treated in the absence (-) or presence (+) of doxycycline for 48 hrs to induce lytic reactivation and assayed by Western blot for ORF50 (upper), ORF45 (middle), or Actin loading control (lower). **(B)** RFP-LANA WT1, WT2, MT1, or MT2 stable iSLK cell lines were assayed by RT-PCR for expression of ORF45, ORF50, or PAN. mRNA was quantified relative to GAPDH.
(TIF)

S6 Fig LANA oligomerization maintains chromosome conformation interaction between latent and lytic control regions. Stable iSLK cells containing either WT or MT RFP-LANA bacmids were assayed by 3C with anchored primer at KSHV latency control region (129360) and interaction pairs at KSHV lytic control regions (69163, or 72974) or negative control (77155). 3C-qPCR relative to actin control is indicated. * p value <0.05, ** p value < .01, and *** p value <0.001 were calculated using two-tailed student t-test.
(TIF)

S7 Fig LANA oligomerization is important for LANA-binding and ORC recruitment to KSHV TR and LANA transcription repression. **A)** Schematic of ChIP-qPCR primer positions with relation to KSHV genes and loci. Red triangles indicate position of CTCF binding. **(B)** ChIP-qPCR analysis of LANA-RFP WTgfp (black) or MTgfp (red) stable iSLK cell lines using antibodies for LANA, ORC2, H3K27me3, or IgG control, as indicated. Primer positions are indicated on the x-axis. * p value < 0.05, ** p value < 0.01 using two-tailed student t-test. **(C)** RT-qPCR analysis of LANA-RFP WTgfp or MTgfp stable iSLK cell lines assaying LANA with (+) or without (-) RT.
(TIF)

S8 Fig LANA oligomerization controls TR chromosome conformation. RFP-LANA WTgfp or MTgfp stable iSLK cell lines were assayed by 3C using anchor primer near TR (position 133872) and assayed at positions indicated on x-axis. 3C-qPCR relative to actin control is indicated. ** p value <0.01 using two-tailed student t-test.
(TIF)

S9 Fig LANA oligomerization is important for viral genome integrity. **(A)** RFP-LANA WTgfp (black) or MTgfp (red) stable iSLK cell lines were analyzed by qPCR for copy number variation using primers spanning KSHV genome, as indicated on X-axis. **(B)** KSHV genome map indicating positions of interest.
(TIF)

S1 Table Primer sequences used for BAC mutagenesis. (XLSX)

S2 Table Primer sequences used for quantification of KSHV DNA. (XLSX)

S3 Table Primer sequences used for 3C PCR. (XLSX)

S1 Movie Original live cell imaging of iSLK with Bac16-RFP-LANA at 40X merged with phase contrast. (MOV)

S2 Movie High resolution, confocal live cell imaging of RFP-LANA during mitotic division in 3 cells. (MOV)

S3 Movie High resolution, confocal live cell imaging of RFP-LANA as shown in M2, with 3D rotation. (MOV)

S1 Data Fasta and annotation text file for Illumina sequencing of the KSHV Bacmid F1037A/F1041A. (TXT)
