# Supplementary figures and images for: LANA oligomeric architecture is essential for KSHV nuclear body formation and viral genome maintenance during latency
Source: PLoS Pathog. 2019 Jan 25;15(1):e1007489. doi: 10.1371/journal.ppat.1007489 (PMC6364946; doi:10.1371/journal.ppat.1007489)

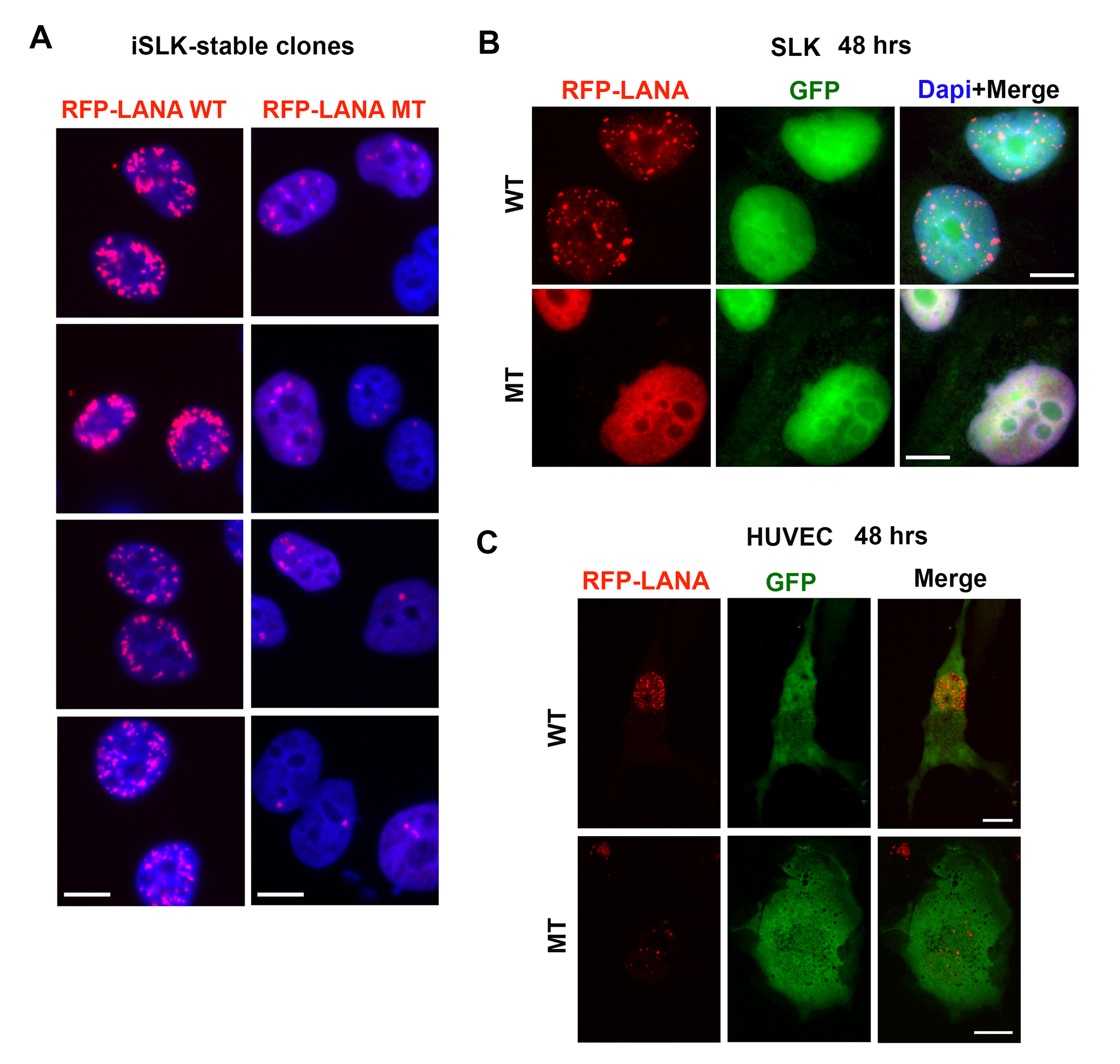

Supplement: S1 Fig — (A) Immunofluorescence analysis of RFP-LANA foci in RFP-LANA WT (left) or MT (right) iSLK stable cells. Representative images showed RFP-LANA (red) and Dapi staining (blue). Scale bar = 10 μm. (B-C) SLK (B) or HUVEC (C) cells were infected with RFP-LANA WT or MT KSHV bacmid virus and immunofluorescence analysis was used to assay RFP-LANA WT (top) and MT (bottom) at 48 hrs post primary infection. Infected cells were shown by GFP (green) and counter-stained by Dapi in merged images. Scale bar = 10 μm. (TIF) [file ppat.1007489.s001.tif]

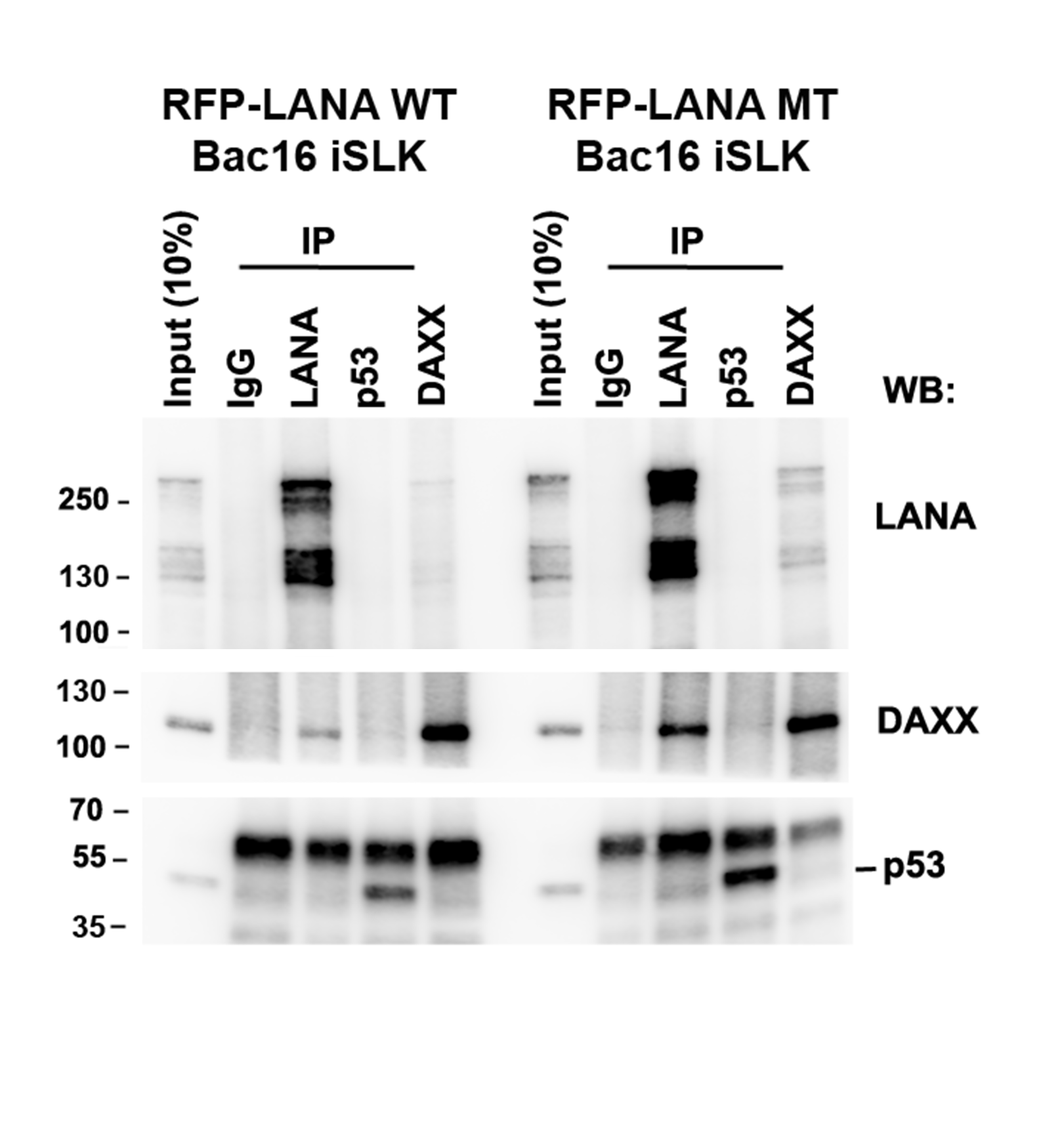

Supplement: S2 Fig — RFP-LANA WT (left) or MT (right) expressed from BAC16 in stable iSLK cells was subject to IP with either IgG, LANA, p53, or DAXX antibodies, and then assayed by Western blot with antibody to LANA (top), DAXX (middle), or p53 (lower). (TIF) [file ppat.1007489.s002.tif]

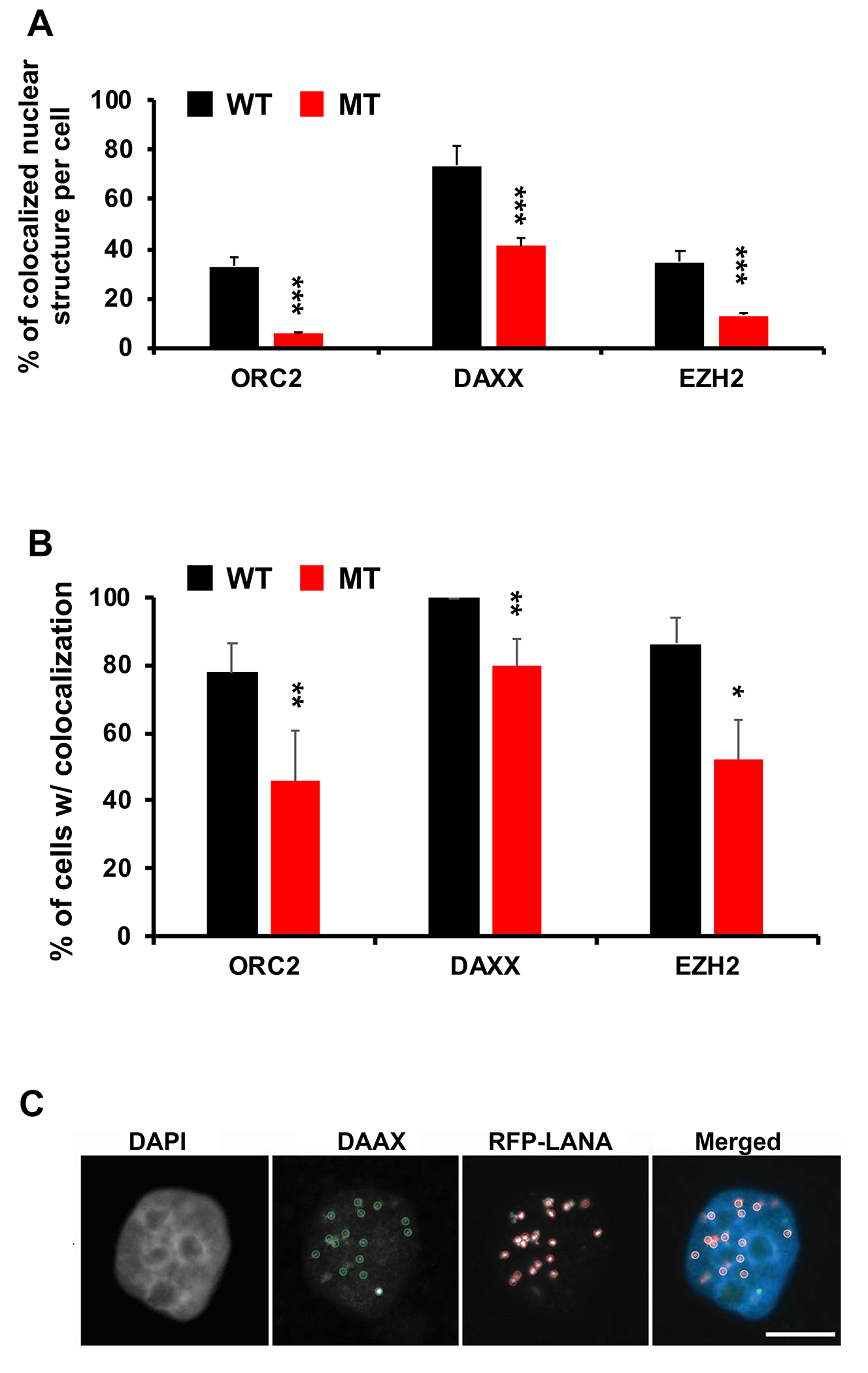

Supplement: S3 Fig — A) The percentage of foci for ORC2, DAXX, or EZH2 that colocalized with LANA bodies for RFP-LANA WT (black) and RFP-LANA MT (red). B) The percentage of cells in the population that display colocalized nuclear structure for ORC2, DAXX, or EZH2 in RFP-LANA WT (black) and RFP-LANA MT (red). Colocalization was determined and quantified using Nikon NIS Elements AR software, version 5.02 using the Spot Detection Tool. **p value < .01, *** p value <0.001 was calculated using two-tailed student t-test. (C) Example of computational method for quantifying colocalization of LANA and DAXX foci. The colored circular outlines indicate the number of Daxx (green) and RFP-LANA (red) foci. The white outlines in the merged image show the number of LANA foci colocalized with Daxx foci. Bar scale = 10um. (TIF) [file ppat.1007489.s003.tif]

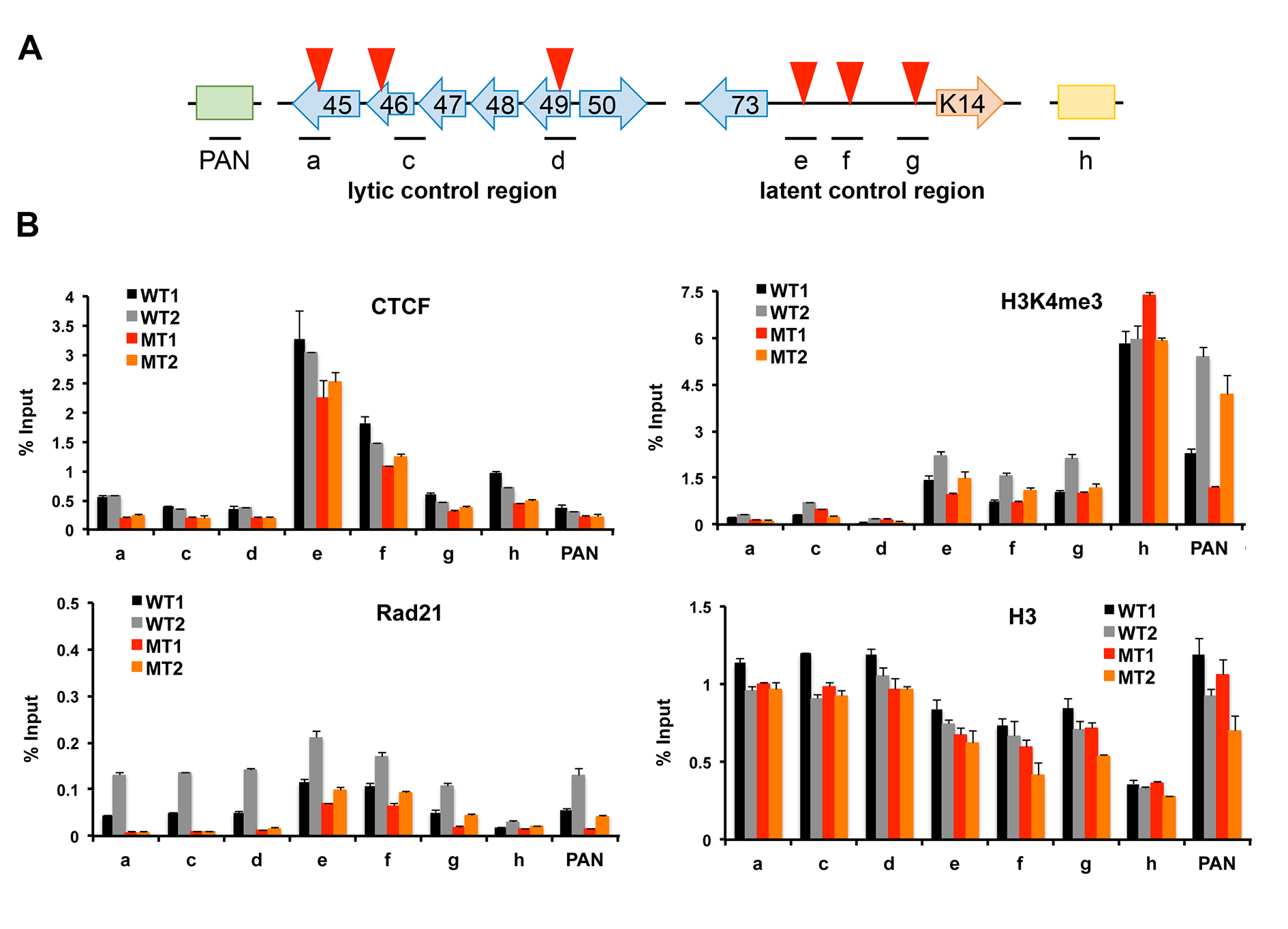

Supplement: S4 Fig — (A) Schematic of ChIP-qPCR primer positions with relation to KSHV genes and loci. Red triangles indicate position of CTCF binding. (B) ChIP-qPCR for LANA-RFP WT1, WT2, MT1, or MT2 stable iSLK cell lines using antibodies for CTCF, H3K4me3, RAD21, and histone H3 as indicated. (TIF) [file ppat.1007489.s004.tif]

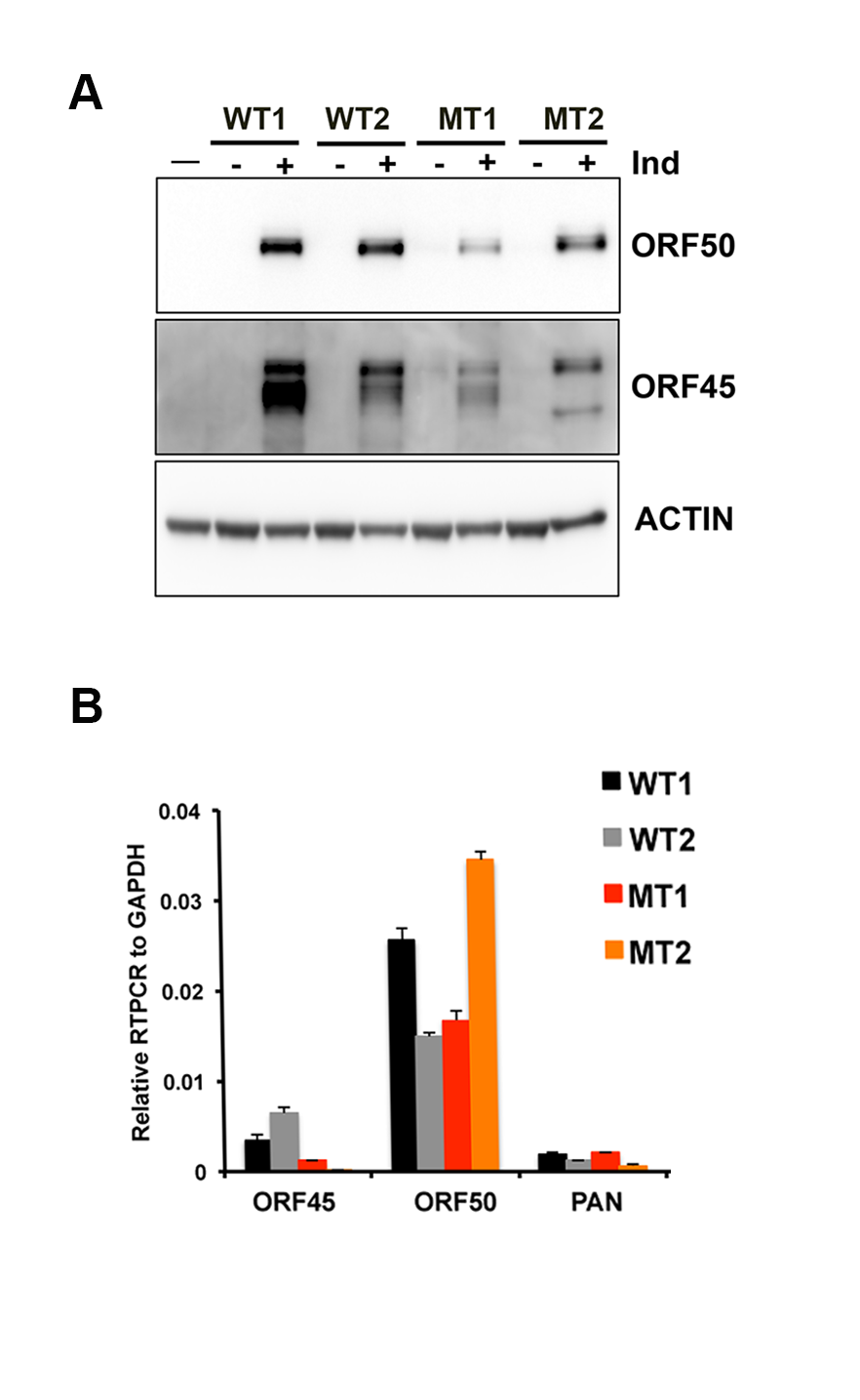

Supplement: S5 Fig — (A) RFP-LANA WT1, WT2, MT1, or MT2 stable iSLK cell lines were treated in the absence (-) or presence (+) of doxycycline for 48 hrs to induce lytic reactivation and assayed by Western blot for ORF50 (upper), ORF45 (middle), or Actin loading control (lower). (B) RFP-LANA WT1, WT2, MT1, or MT2 stable iSLK cell lines were assayed by RT-PCR for expression of ORF45, ORF50, or PAN. mRNA was quantified relative to GAPDH. (TIF) [file ppat.1007489.s005.tif]

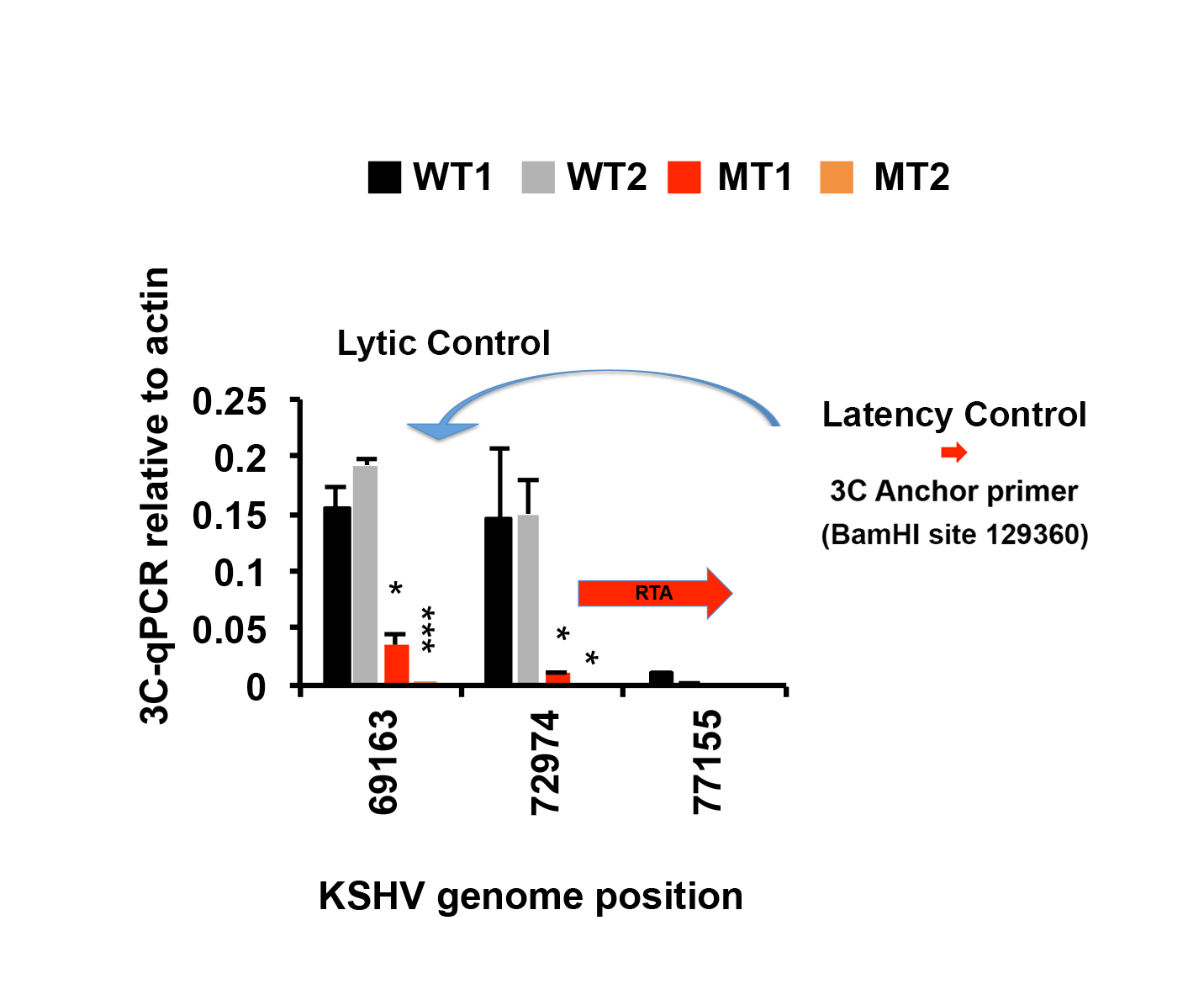

Supplement: S6 Fig — Stable iSLK cells containing either WT or MT RFP-LANA bacmids were assayed by 3C with anchored primer at KSHV latency control region (129360) and interaction pairs at KSHV lytic control regions (69163, or 72974) or negative control (77155). 3C-qPCR relative to actin control is indicated. * p value <0.05, ** p value < .01, and *** p value <0.001 were calculated using two-tailed student t-test. (TIF) [file ppat.1007489.s006.tif]

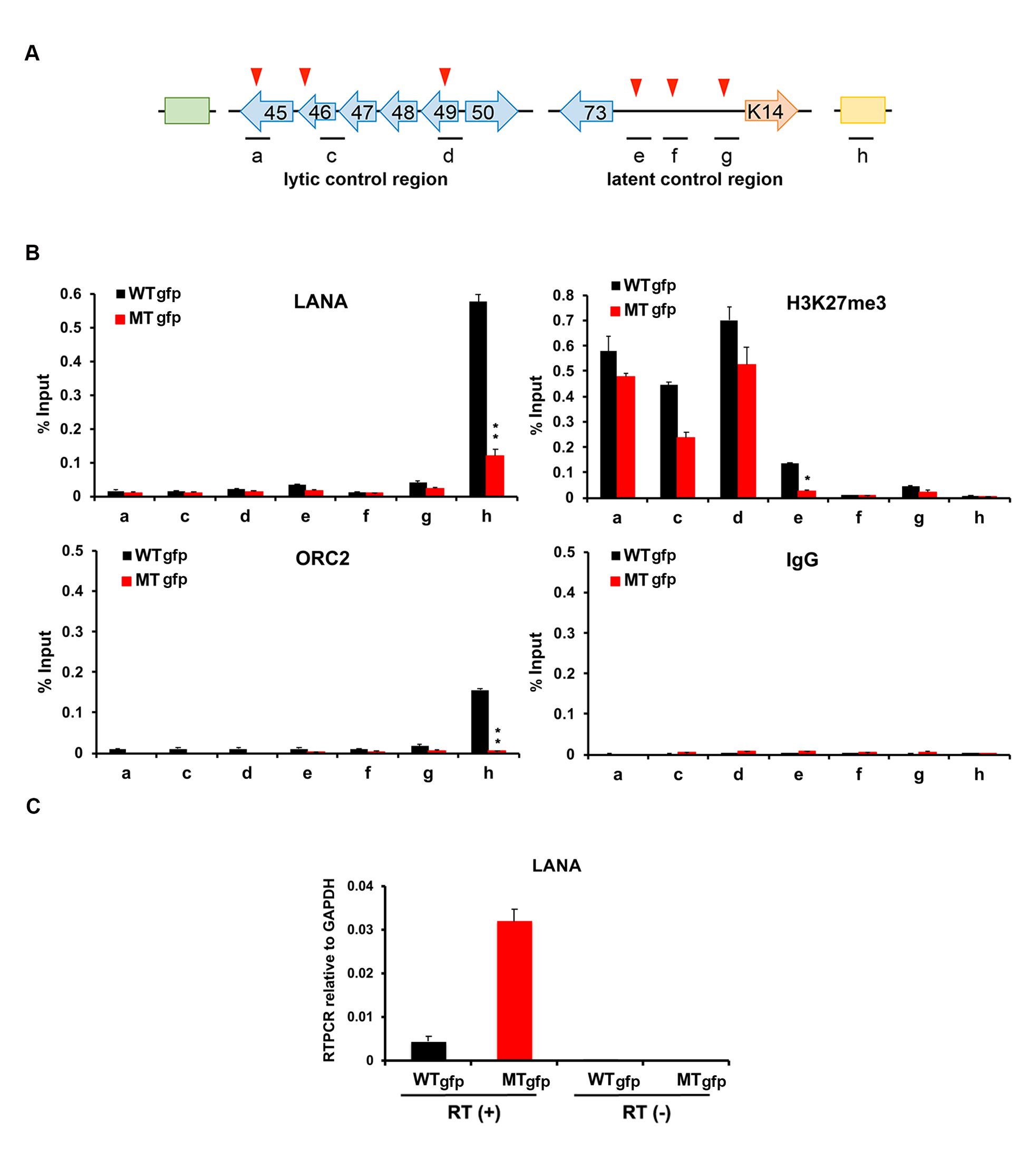

Supplement: S7 Fig — A) Schematic of ChIP-qPCR primer positions with relation to KSHV genes and loci. Red triangles indicate position of CTCF binding. (B) ChIP-qPCR analysis of LANA-RFP WTgfp (black) or MTgfp (red) stable iSLK cell lines using antibodies for LANA, ORC2, H3K27me3, or IgG control, as indicated. Primer positions are indicated on the x-axis. * p value < 0.05, ** p value < 0.01 using two-tailed student t-test. (C) RT-qPCR analysis of LANA-RFP WTgfp or MTgfp stable iSLK cell lines assaying LANA with (+) or without (-) RT. (TIF) [file ppat.1007489.s007.tif]

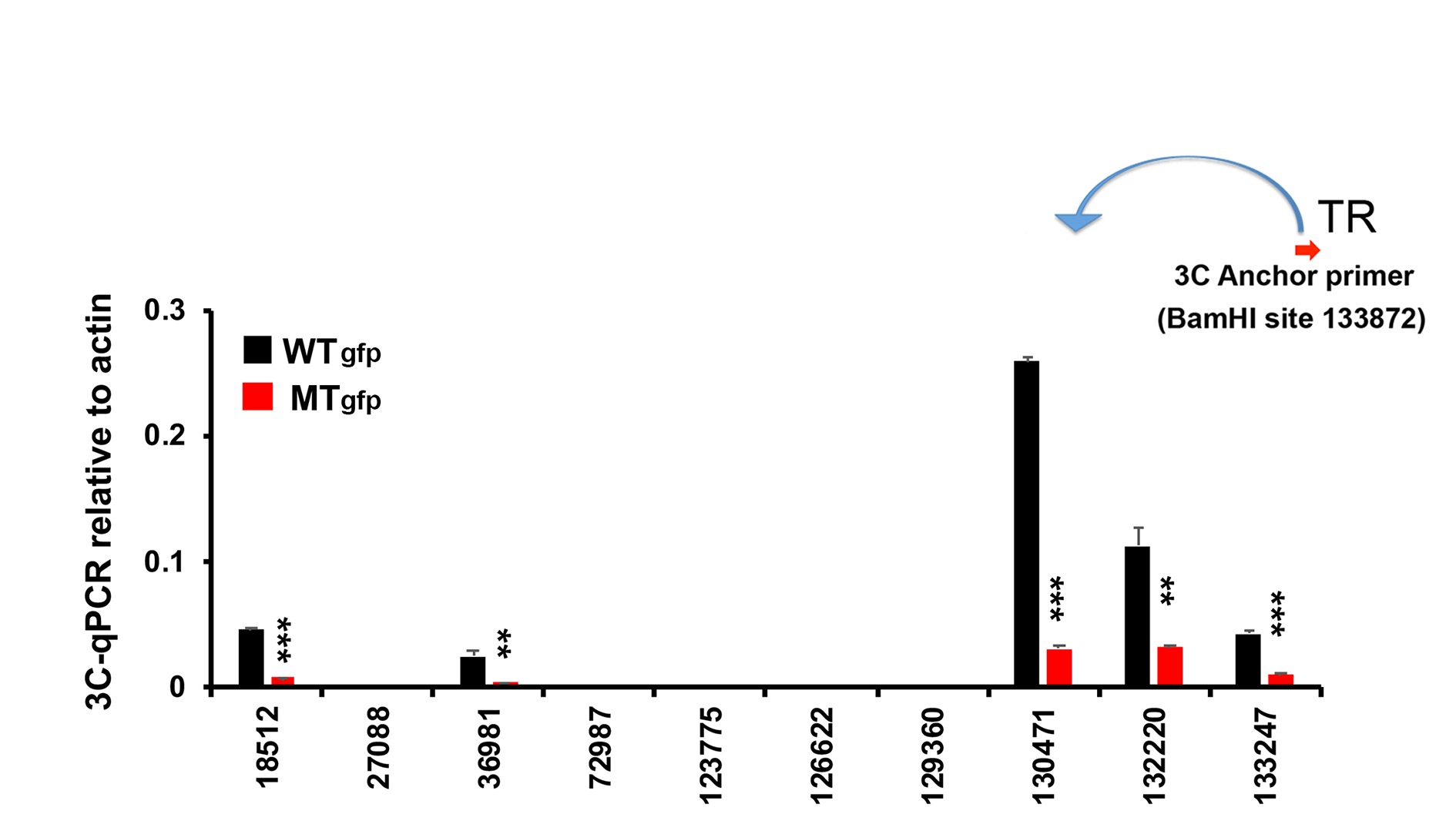

Supplement: S8 Fig — RFP-LANA WTgfp or MTgfp stable iSLK cell lines were assayed by 3C using anchor primer near TR (position 133872) and assayed at positions indicated on x-axis. 3C-qPCR relative to actin control is indicated. ** p value <0.01 using two-tailed student t-test. (TIF) [file ppat.1007489.s008.tif]

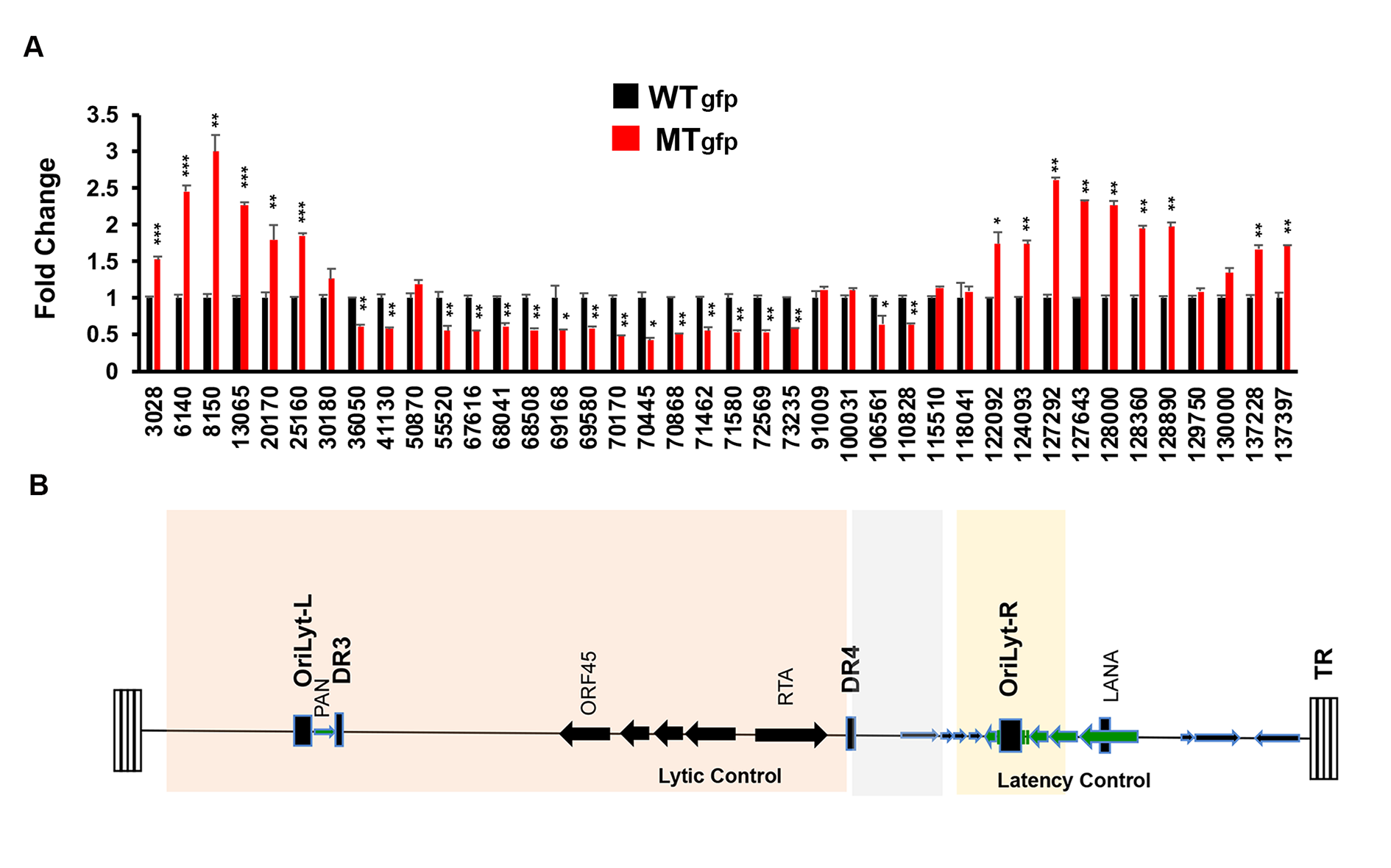

Supplement: S9 Fig — (A) RFP-LANA WTgfp (black) or MTgfp (red) stable iSLK cell lines were analyzed by qPCR for copy number variation using primers spanning KSHV genome, as indicated on X-axis. (B) KSHV genome map indicating positions of interest. (TIF) [file ppat.1007489.s009.tif]
